# Predicting the Stability of Base‐mediated C─H Carboxylation Adducts Using Data Science Tools

**DOI:** 10.1002/anie.202504934

**Published:** 2025-11-19

**Authors:** Maike Eckhoff, Shubham Deolka, Aleria Garcia‐Roca, Lilly Meynberg, Liudmila Seidel, Matthew S. Sigman, Jonny Proppe

**Affiliations:** ^1^ TU Braunschweig Institute of Physical and Theoretical Chemistry Gauss Str 17 38106 Braunschweig Germany; ^2^ University of Utah Department of Chemistry Salt Lake City Utah 84112 USA

**Keywords:** C1 building block, Carbon dioxide, C─H functionalization, Machine learning, Thermodynamic stability

## Abstract

Base‐mediated C–H carboxylation is a versatile pathway for utilizing carbon dioxide (CO_2_) as a C1 building block in organic synthesis. However, CO_2_ constitutes a notorious thermodynamic sink, which restricts this approach to activated or intrinsically reactive nucleophiles. To qualitatively assess the stability of CO_2_ adducts, we present a computational approach that integrates quantum chemistry with statistical modeling to build a predictive workflow. The target property is the CO_2_ affinity, specifically the negative Gibbs free reaction energy. This predictive workflow has been applied to 60 novel carbon‐centered nucleophiles, suggesting reactions that yield stable carboxylation adducts. The results have been validated through experimental methods for five carbanions, which include three stable and two unstable adducts in DMSO according to our predictions. In addition, we examined two further carbanions that were suggested to form stable CO_2_ adducts in DMSO, to further assess the experimental protocol and broaden its scope to structurally distinct motifs.

## Graphical abstract

A computational approach integrating quantum chemistry and machine learning enables the prediction of CO_2_–adduct stability. Model interpretability and experimental validation highlight its utility for designing base‐mediated C–H carboxylation reactions.

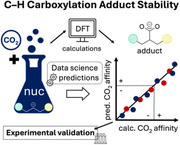

Carbon dioxide (CO_2_) serves as a valuable C1 building block in organic synthesis.^[^ ^1^, ^2^, ^3^, ^4^, ^5^ ^]^ Carboxylation products find numerous applications, including CO_2_‐binding strategies^[^ ^6^, ^7^, ^8^, ^9^ ^]^ and serving as key components in pharmaceuticals, particularly in prodrugs, as esters and amides.^[^ ^10^ ^]^ Of the many synthetic tactics for incorporating CO_2_ into molecules, C─H carboxylation is particularly relevant due to its high atom and step economy.^[^ ^11^, ^12^ ^]^ When carrying out base‐mediated C─H carboxylation under mild and transition‐metal‐free conditions, the resulting processes are potentially both more environmentally friendly and cost‐efficient.^[^ ^13^ ^]^ Previous studies (Scheme 1a) have highlighted base‐mediated carboxylation reactions promoted by Cs_2_CO_3_ for electron‐deficient aromatic heterocycles, as reported by Vechorkin et al.,^[^ ^14^ ^]^ Fenner and Ackermann demonstrated that these reactions are achievable using KO*t*Bu as the stoichiometric base.^[^ ^15^ ^]^ The resulting highly nucleophilic carbanion facilitates the subsequent CO_2_ capture step at low to moderate temperatures and atmospheric CO_2_ pressure. In the work of Felten et al., base‐mediated carboxylation reactions of azoles were activated and stabilized by silyl triflate reagents.^[^ ^16^ ^]^

**Scheme 1:**
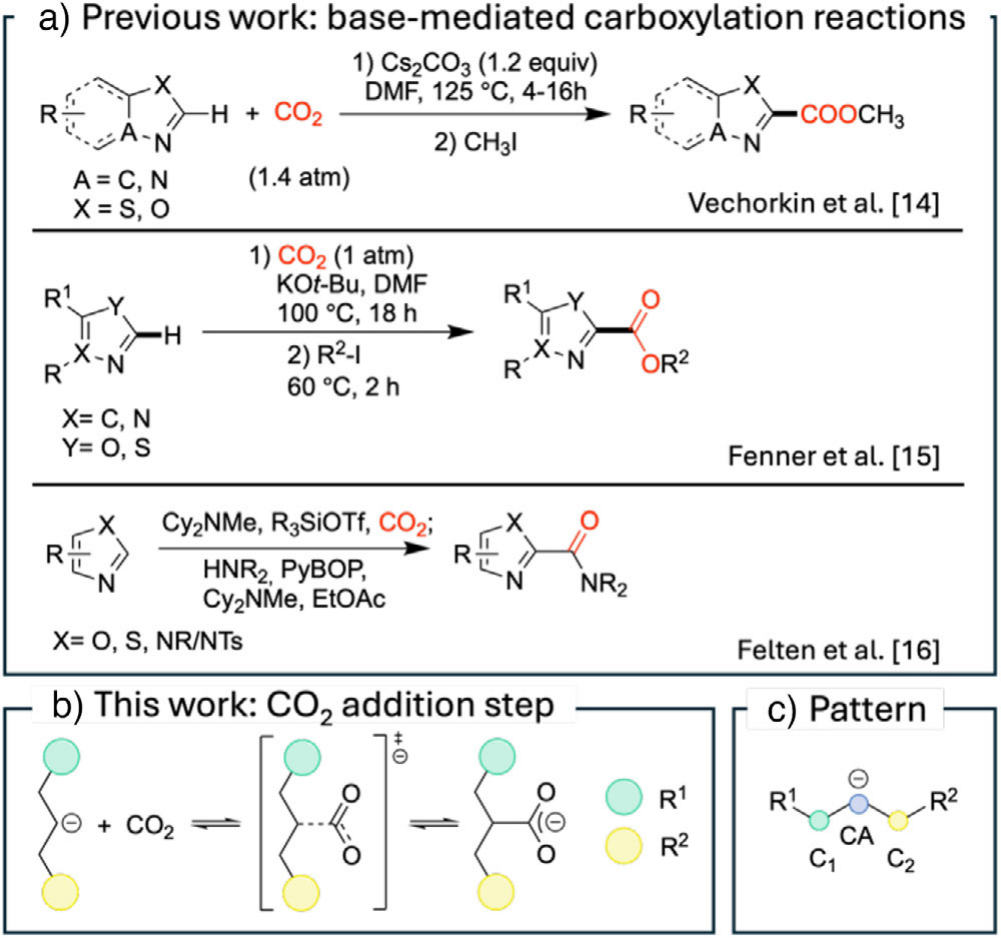
a) Previous work on base‐mediated C─H carboxylation reactions.^[^ ^14^, ^15^, ^16^ ^]^ b) Carboxylation step considered in this study based on the experimental protocol by Fenner and Ackermann.^[^ ^15^ ^]^ c) Nucleophilic structure pattern considered in this work. The carbanionic site CA has two carbon neighbors, C_1_ and C_2_.

However, an underlying aim in this area is to determine when carbanions will form stable CO_2_ adducts as they are generally unstable due to the high thermodynamic stability of CO_2_.^[^ ^17^ ^]^ While a reactive nucleophile can facilitate adduct formation, stabilizing this adduct poses a different challenge, although kinetic barriers may correlate with reaction energies under certain conditions.^[^ ^18^ ^]^ Recent studies have explored various approaches to address these thermodynamic challenges. For instance, Li et al. successfully synthesized a carboxylation product using indene through an acidic workup.^[^ ^19^ ^]^ Additionally, employing reagents that further react with the carboxylation adducts has proven effective to circumvent decomposition.^[^ ^14^, ^15^, ^16^ ^]^ To achieve a comprehensive understanding of the carboxylation process, it is also essential to consider the kinetics of carboxylating carbanions as well. Recent studies^[^ ^19^, ^20^ ^]^ have indicated that CO_2_ is suitable to react with a diverse set of nucleophiles in DMSO, including succinimide^[^ ^21^ ^]^ or piperidine.^[^ ^22^ ^]^ An overarching goal would be to establish structural guidelines through the development of a predictive model for when stable CO_2_ adducts will form. Such models could guide synthetic chemists in CO_2_ capture and valorization while also providing fundamental knowledge. Toward this goal, we report herein a predictive model of the formation of stable CO_2_ adducts arising through base‐mediated C─H carboxylation focused on the specific reaction step depicted in Scheme 1b. The resultant model was used to both understand the structural features required to form stable adducts and was applied prospectively to identify new structures that were validated experimentally.

As the first step, we generated a dataset based on 31 structurally diverse nucleophiles (Scheme 2a). These nucleophiles were selected for their kinetic suitability to react with CO_2_, as indicated by Mayr and Patz's reactivity scale.^[^ ^19^, ^20^ ^]^ To produce physical organic molecular descriptors, conformer ensembles were obtained using the GFN2‐xTB^[^ ^23^ ^]^ method implemented in CREST,^[^ ^24^, ^25^ ^]^ followed by density functional theory (DFT) calculations with Gaussian.^[^ ^26^ ^]^ Structure optimizations were performed with PBE‐D3(BJ)/def2‐TZVPD^[^ ^27^, ^28^, ^29^, ^30^, ^31^ ^]^ including implicit solvation using the SMD model^[^ ^32^ ^]^ for DMSO. From the resultant structures, a range of steric and electronic descriptors were extracted.^[^ ^33^, ^34^, ^35^ ^]^ For each descriptor, ensemble‐based values were collected, including the minimum (min), maximum (max), and the lowest‐energy conformer (lowE) value, as well as the Boltzmann‐weighted average (Boltz) values of the ensemble. To train a model, the affinity of CO_2_ for binding to the carbon‐centered nucleophile (see structure pattern in Scheme 1c) was assessed by computing the negative Gibbs free reaction energy as the target property (CO_2_A_calc_) at a higher computational level using B3LYP^[^ ^36^, ^37^ ^]^ as exchange–correlation functional instead of PBE (see computational details and benchmark as well as modeling details in Sections S1–S5).

**Scheme 2:**
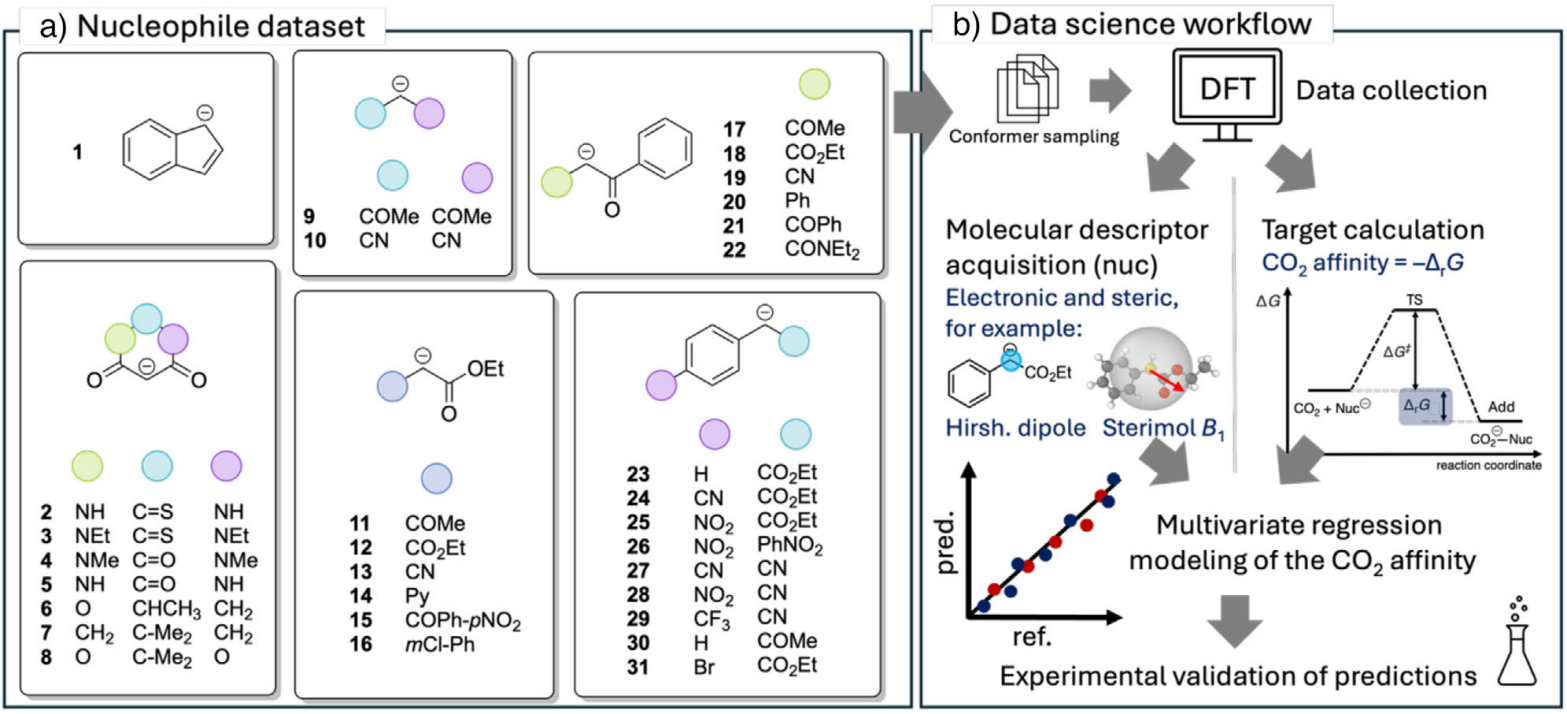
a) Dataset of 31 nucleophiles used for reference. The nucleophiles all exhibit the same structure pattern (Scheme 1c) and were kinetically characterized by the Mayr/Ofial group.^[^ ^39^ ^]^ b) Overview of the data science workflow applied in this work: Initially, PBE calculations were performed on conformer ensembles to obtain computational data for the nucleophiles in the dataset. The CO_2_ affinity is then evaluated using B3LYP. For the nucleophiles, steric and electronic molecular descriptors from physical organic chemistry were extracted and subsequently applied to multivariate linear regression modeling. Finally, the resulting model was validated experimentally.

Initially, a multivariate linear regression (MLR) model was developed by correlating the CO_2_A_calc_ to the molecular features of the nucleophiles.^[^ ^34^, ^38^ ^]^ Model performance was measured by statistical metrics, including the coefficient of determination (*R* ^2^) and the mean absolute error (MAE, in kcal mol^−1^) for both training and test sets. A leave‐one‐out (LOO) analysis and *k*‐fold cross‐validation (*k* = 5) were performed to evaluate the overall robustness of the model (see SI for details).

The best three‐parameter MLR model (Scheme 3a) performs well on the test set (*R* ^2^ = 0.95) and in five‐fold cross‐validation (*R* ^2^ = 0.97). This model is based on two electronic descriptors and one steric descriptor: the energy of the highest occupied molecular orbital (HOMO), ε_HOMO_, the Hirshfeld dipole moment at the carbanionic site (CA, see Scheme 1b), and the buried Sterimol^[^ ^40^ ^]^ *B*_1_ value defined at CA and C_1_/C_2_ (see Scheme 3b). In Scheme 3c, the electronic properties are plotted against each other and the magnitude of the steric parameter is reflected by the size of datapoints. The CO_2_ affinity is visualized as a heat map. The plot reveals a clear trend: the lower ε_HOMO_, the higher the CO_2_ affinity. A threshold of *ε*_HOMO_ = –0.140 E_h_ can be identified for a CO_2_ affinity of approximately zero. The two additional parameters improve the model, as *ε*_HOMO_ alone does not fully capture the observed relationship. Two nucleophiles appear to be outliers: **22** (classified as false negative) and **1** (classified as false positive). This highlights a limitation of the model: both nucleophiles feature uncommon structural motifs that are not well captured by linear regression.

**Scheme 3:**
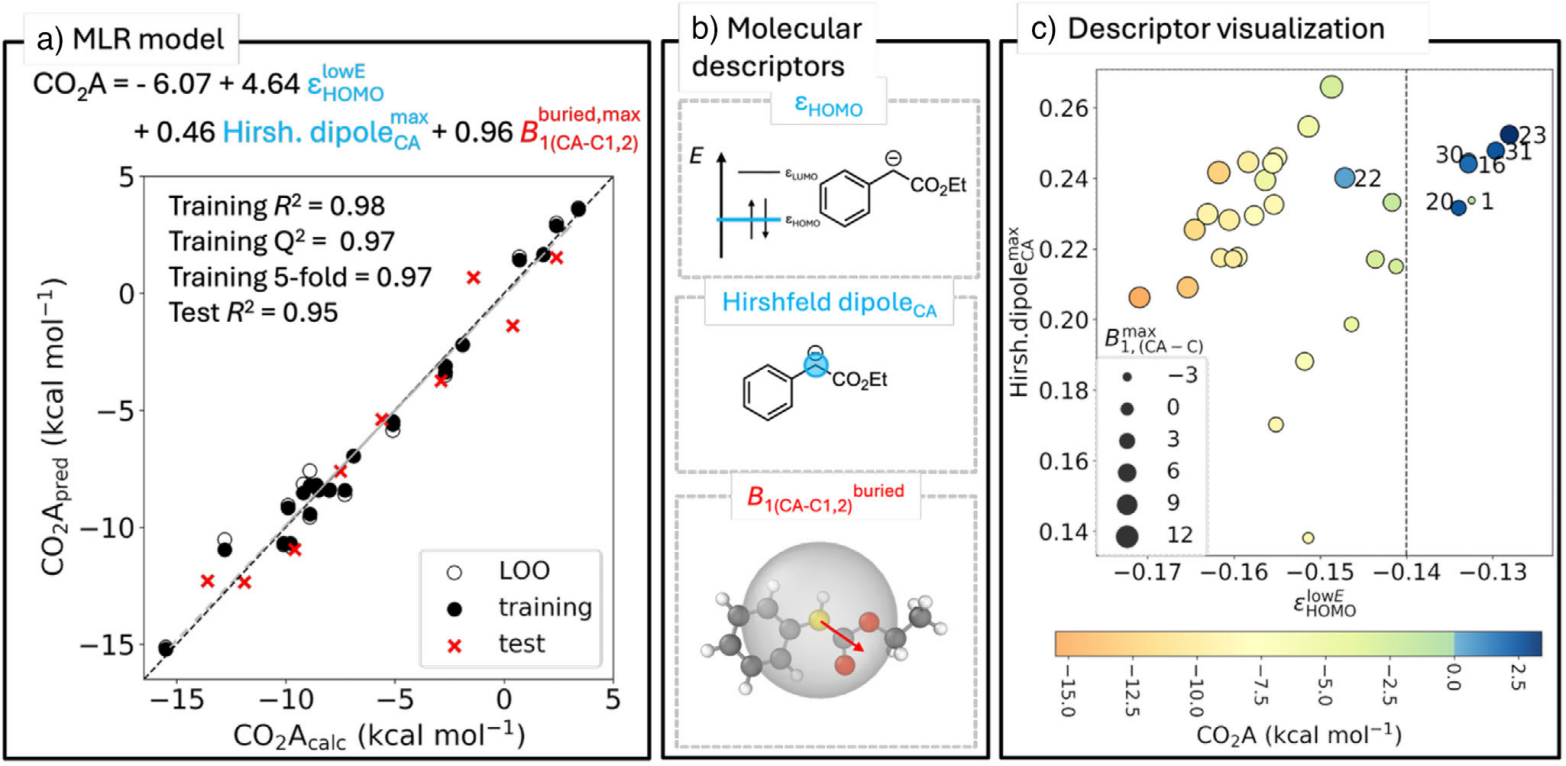
a) MLR model for predicting CO_2_ affinities (CO_2_A) by three parameters: The electronic descriptors (blue) represent the energy of the highest unoccupied molecular orbital (HOMO), *ε*_HOMO_, and the Hirshfeld dipole moment at the carbanionic site CA, respectively. The steric descriptor is the buried Sterimol *B*_1_ value defined at CA and C_1_/C_2_. b) Visualization of molecular descriptors incorporated in the MLR model. c) Descriptor visualization. Both electronic contributions are plotted against each other. The steric contribution is reflected by the size of circles, and the color encodes the CO_2_ affinity.

The model parameters provide valuable insight into the CO_2_ addition step. A higher *ε*_HOMO_ indicates a more favorable electron transfer to CO_2_, facilitating bond formation in the adduct. Additionally, a larger Hirshfeld dipole moment at the carbanionic site suggests a stronger electrostatic attraction with CO_2_. The buried Sterimol *B*_1_ value represents the nucleophile's bulkiness: as it increases, steric shielding enhances adduct stabilization.

In the next step, we constructed an automated and user‐friendly workflow that integrates both the classification and MLR models to screen nucleophiles and focus the search for potentially stable CO_2_ adducts (see Scheme 4 and https://git.rz.tu‐bs.de/proppe‐group/co2_affinity_prediction). To initiate the workflow, 60 potential nucleophiles were combinatorially designed based on core structural features found in nucleophiles forming the most stable adducts of the initial dataset (see Scheme 5a). The only required input is a set of SMILES strings. To quickly assess the structures after an initial DFT calculation but before full computational analysis, they were screened based on the *ε*_HOMO_ threshold. Of those evaluated, 39 nucleophiles met the criterion, and their CO_2_ affinities were predicted using the MLR model (see Scheme 5b). The predicted CO_2_ affinities of the top candidates identified for stable product formation are summarized in Table 1. Of these, five nucleophiles—**11**, **12**, **38**, **39**, **41**—were subjected to experimental validation. To balance the validation, three nucleophiles (**38**, **39**, and **41**) were selected that have high predicted CO_2_ affinities, while two (**11** and **12**) were calculated to have negative CO_2_ affinities in DMSO at 20 °C. Consistent with the predictions, nucleophiles **38**, **39**, and **41** reacted with CO_2_ to form the desired adducts (RCOO^−^) as detected by direct injection HRMS analysis (see the ESI). Nucleophiles **11** and **12** did not form adducts in agreement with our model. However, NMR identification of the detected adducts in DMSO was not possible, presumably due to poor stability in the experimental process, consistent with results from the Mayr/Ofial group (see the ESI for details).^[^ ^19^ ^]^ Therefore, we evaluated different conditions, wherein we found that when toluene was used as solvent at 80 °C that all three of the positively predicted nucleophiles yielded the desired carboxylated products (38% for **38**, 35% for **39**, and 33% for **41**, confirmed by ^1^H NMR spectroscopy and HRMS (Table 1)). Quantum‐chemical calculations under the experimental conditions (toluene, 80 °C) likewise reproduced the observed outcome, predicting three stable and two unstable adducts. The identification of products in toluene could be attributed to the better stability of in‐situ generated carbanions.

**Scheme 4:**
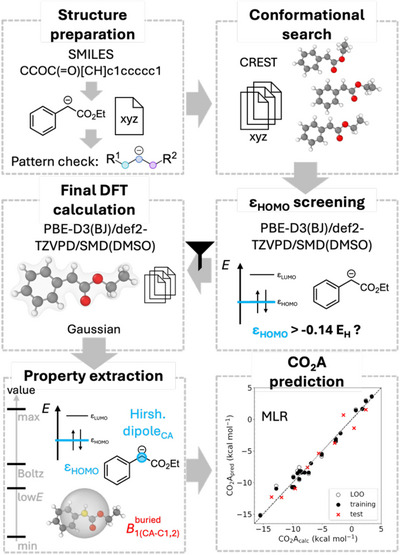
Workflow steps involved in the automated prediction of CO_2_ affinities. Initially, SMILES strings are converted into three‐dimensional structures, while simultaneously assessing the nucleophilic pattern. Following a conformational search, DFT calculations are performed to filter nucleophiles based on their HOMO energy. Nucleophiles with *ε*_HOMO_ values above −0.140 E_h_ enter a second round of DFT calculations after which the MLR properties are extracted. Finally, the CO_2_ affinity is predicted by the MLR model, and the results are visualized for clarity.

**Scheme 5:**
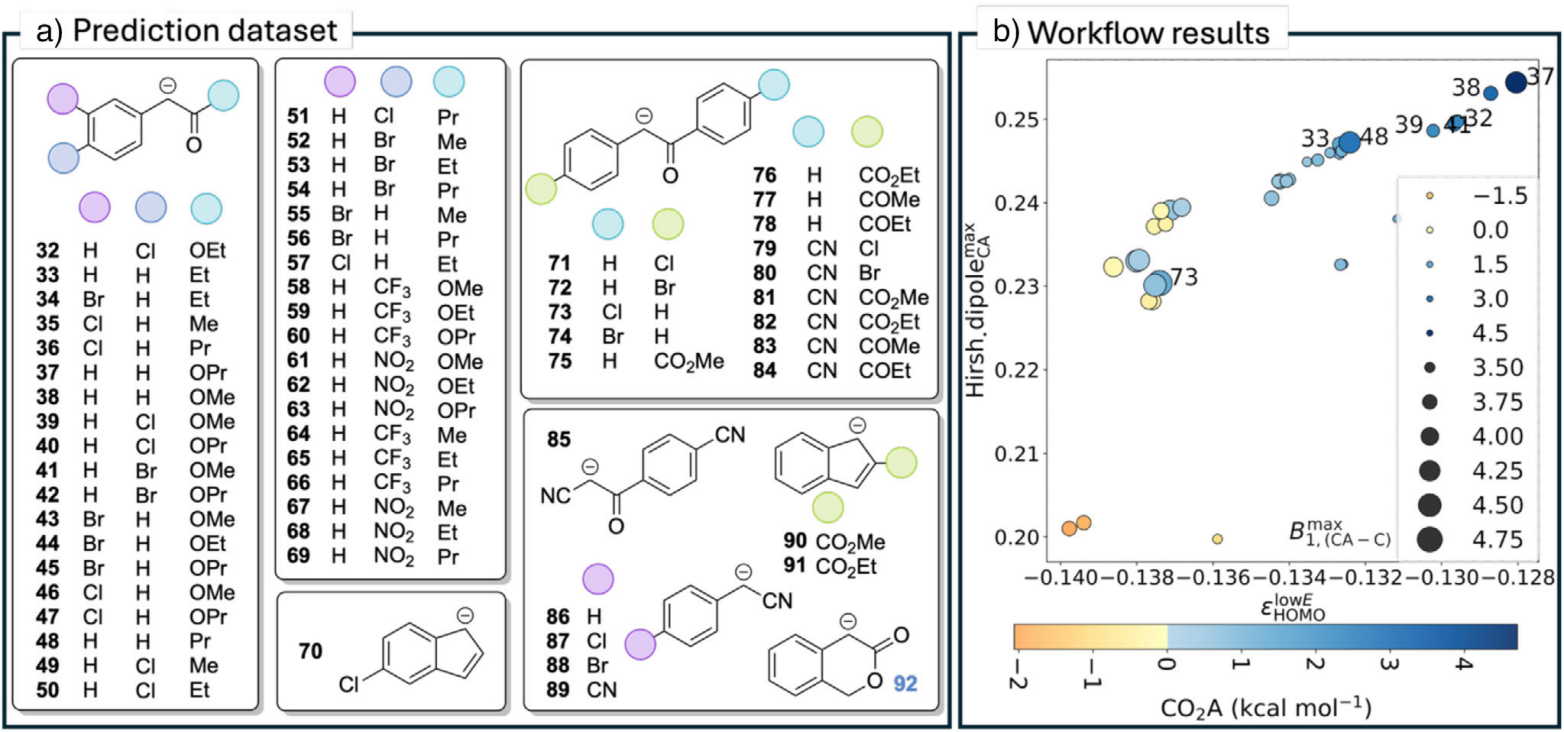
a) Screening dataset with 60 nucleophiles. b) CO_2_ affinity predictions for the nucleophiles coming out of the automated prediction workflow.

**Table 1:** Predictions^a)^ for the eight most stable adducts listed in descending order, including *ε*_HOMO_ (in E_h_) and CO_2_ affinities (in kcal mol^−1^). The observed yield was obtained by measuring ^1^H NMR using 1,3,5‐trimethoxybenzene as internal standard.

Nucleophile	*ε*_HOMO_	CO_2_ affinity	Detected in DMSO	Yield in Toluene
**1**	−0.137	−1.32	Yes*	
**37**	−0.128	+4.72		
**38**	−0.129	+3.12	Yes	38 %
**48**	−0.132	+2.89		
**32**	−0.130	+2.63		
**41**	−0.130	+2.56	Yes	33 %
**92**	−0.137	+2.25	Yes	
**39**	−0.130	+1.85	Yes	35 %
**33**	−0.133	+1.53		
**73**	−0.137	+1.47		
**12**	−0.153	−2.7^b)^	No	2.6 %
**11**	−0.150	−5.6^b)^	No	5.9 %

^a)^ The two different solvents used for model development and binary experimental response (DMSO) and yields in (toluene) were compared to ensure reliable validation results. Although the stability in toluene decreases relative to DMSO, the overall trend remains consistent, and both groups of nucleophiles (**11**, **12** versus **38**, **39**, **41**) are clearly separated from each other (see details in Section S1). *Yield for substrate **1** is 65%.

^b)^ CO_2_ affinity obtained from quantum chemical calculations.

Next, to further test the transferability of the predictions and to assess the robustness of the experimental protocol (DMSO, 20 °C), we examined two additional secondary carbanions, **1** (indene) and **92** (isochromanone). Compound **1** was a particularly interesting case, as quantum‐chemical calculations predicted it to be unstable although the HOMO energy is indicative of a stable structure (Table 1), whereas the ML workflow suggested stability. Prior results from the Mayr/Ofial group likewise indicated that **1** forms a stable adduct, which we were able to confirm experimentally. Compound **92**, which differs structurally from the other nucleophiles and was suggested by the Ofial group in personal communication, was also tested. Here as well, a stable adduct was observed. Upon deprotonation with KO^t^Bu and subsequent reaction with CO_2_ in DMSO, the desired protonated adducts were successfully identified (see Table 1 and the Supporting Information).

To further contextualize these results, we established a correlation between kinetic barriers and reaction energies using Mayr's nucleophilicity scale to estimate the CO_2_ affinity of carbanions and potentially other nucleophiles.^[^ ^18^ ^]^ Indeed, we observed a modest correlation of the reactivity, expressed as nucleophilicity *N*, with the CO_2_ affinity (here, *R* ^2^ = 0.74). In short, higher nucleophilicity corresponds to lower kinetic barriers and more stabilized products, leading to increased CO_2_ affinity (see details in Section S8). Because the *N* parameter is available only for nucleophiles that have been kinetically characterized and are listed in Mayr's Reactivity Database, it cannot be incorporated into the model for design purposes. However, existing *N* values serve well as benchmarks.

In conclusion, we have developed a workflow for assessing the stability of CO_2_ adducts formed with carbon‐centered nucleophiles. Our approach employs a three‐parameter multivariate linear regression model that integrates both electronic and steric factors to accurately estimate the CO_2_ affinity. While the current study focused on a set of well‐characterized nucleophiles to establish a robust and reproducible modeling framework, the workflow is readily transferable to larger and more diverse libraries, including literature‐derived nucleophile candidates, as data availability and consistency improve. Future extensions of this framework are aimed at uncovering design‐relevant structural motifs that can guide the selection and modification of nucleophiles across a broader chemical space. Experimental validation of stable and unstable adduct formation reinforces the reliability of our model. The prediction workflow is user‐friendly for users with some experience in running Python‐based workflows that interface with quantum chemistry software, and requires only SMILES strings as input, making it a potentially useful tool for those planning CO_2_ addition experiments. By integrating the thermodynamic stability findings from this study with kinetic data from previous research, we can determine nucleophiles that are likely to successfully undergo carboxylation reactions from both kinetic and thermodynamic perspectives. This insight facilitates designing novel prodrugs and carbamates, thereby advancing pharmaceutical development and improving strategies for CO_2_ binding.

## Supporting Information

The authors have cited additional references within the Supporting Information.^[^ ^41^, ^42^, ^43^, ^44^, ^45^, ^46^, ^47^ ^]^ Computational and experimental details are provided. The computationally optimized structures, and statistical modeling scripts of this communication are available at https://git.rz.tu‐bs.de/proppe‐group/co2_affinity_prediction.

## Conflict of Interests

The authors declare no conflict of interest.

## Supporting information



Supporting Information

## Data Availability

The authors have cited additional references within the Supporting Information. Computational and experimental details are provided. The computationally optimized structures, and statistical modeling scripts of this communication are available at https://git.rz.tu‐bs.de/proppe‐group/co2_affinity_prediction.
