# Supplementary material for: Predicting the Stability of Base‐mediated C─H Carboxylation Adducts Using Data Science Tools
Source: Angew Chem Int Ed Engl. 2025 Nov 19;65(2):e04934. doi: 10.1002/anie.202504934 (PMC12790316; doi:10.1002/anie.202504934)
Supplement: Supplementary file 1 — Supporting Information [file ANIE-65-e04934-s001.pdf]

Supporting Information for Predicting the Stability of Base-mediated C–H Carboxylation Adducts using Data Science Tools

Maike Eckhoff,[a,b] Shubham Deolka[b] ,Aleria Garcia-Roca,[[b] Lilly Meynberg,[a] Liudmila Seidel,[a] Matthew S. Sigman,*[b] and Jonny Proppe,*[a]

[a] Dr. M. Eckhoff, L. Meynberg, L. Seidel, Prof. Dr. J. Proppe
Technische Universität Braunschweig
Institute of Physical and Theoretical Chemistry
Gauss Str. 17, 38106 Braunschweig, Germany
E-mail: j.proppe@tu-braunschweig.de

[b] Dr. S. Deolka, Dr. A. Garcia-Roca, Prof. Dr. M. S. Sigman
University of Utah
Department of Chemistry
Salt Lake City, Utah 84112, United States
Email: matt.sigman@utah.edu

More supporting information can be accessed through the project-related Git-Lab repository: https://git.rz.tu-bs.de/proppe-group/co2_affinity_prediction.

Section S1: Computational methods for target calculations (CO_2 affinity)

CREST (version 2.12)[1,2] was utilized to search for the conformer ensemble for each molecule (CO_2, nucleophile, CO_2–nucleophile adduct) using the GFN2-xTB[3] method. Full structure optimizations for the lowest-energy conformers were then performed with Gaussian (version 16, revision C01)[4], employing the hybrid exchange–correlation functional B3LYP[5,6] with the D3-type dispersion correction with Becke–Johnson damping function[7,8] (D3(BJ)) and an ultrafine integration grid. Tight convergence criteria were applied and the def2-TZVPD basis set[9,10] was utilized, which was generated using the Basis Set Exchange program[11]. The basis set has been benchmarked in Eckhoff et al.[12] for similar reactions. Implicit solvation (SMD model[13]) was employed with dimethyl sulfoxide (DMSO) as the solvent. Harmonic frequency calculations at 293.15 K were conducted to confirm the convergence of the structures. Goodvibes[14] was used to apply the quasi-harmonic correction to the vibrational entropy[15] computed by Gaussian.

In this study, the stability of the CO_2–nucleophile adduct is defined as the negative Gibbs free energy of reaction, termed CO_2 affinity (CO_2A) calculated by equation 7 from reference[16]. As validation experiments utilized toluene instead of DMSO (see Section S7 for experimental details), the impact on the optimized structures and related properties were investigated. A recent study by Liu et al.[17] (Figure 5) demonstrated that while the solvent effects are quantitatively different, a qualitatively similar trend was observed. Additionally, Figure S1 presents test results for 9 reactions comparing stability in DMSO and toluene, yielding satisfactory results with an RMSE of 0.59 and R^2 of 0.97, indicating a strong correlation between the results obtained in both solvents. Notably, the stability is qualitatively lower in toluene compared to DMSO.

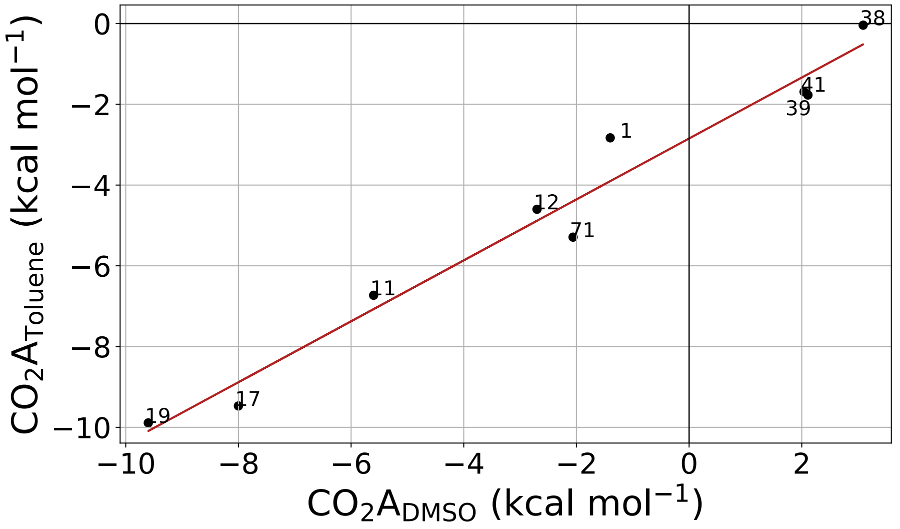

Figure S1: Comparison of CO_2 affinities calculated in DMSO and toluene for nine reactions.

Section S2: Computational methods for descriptor calculations

CREST (version 2.12)[1,2] was utilized to search for the conformer ensemble for each nucleophile using the GFN2-xTB[3] method. Full structure optimizations for the complete conformer ensembles (structures within 6.00 kcal/mol of the lowest-energy conformer) were then performed with Gaussian (version 16, revision C01)[4], employing the hybrid exchange–correlation functional PBE[18] with the D3-type dispersion correction with Becke–Johnson damping function[7,8] (D3(BJ)) and an ultrafine integration grid. Tight convergence criteria were applied and the def2-TZVPD basis set[9,10] was utilized. Implicit solvation (SMD model[13]) was employed with dimethyl sulfoxide (DMSO) as the solvent. Harmonic frequency calculations at 293.15 K were conducted to confirm the convergence of the structures. Natural bond orbital (NBO) analysis was conducted with the NBO program (version 7.0)[19] and NMR shifts were calculated using the gauge-invariant atomic orbitals (GIAO) method[20].

Section S3: Molecular descriptors

Goodvibes[14] was used to apply the quasi-harmonic correction to the vibrational entropy[15] calculated by Gaussian. For the acquisition of molecular descriptors, nucleophiles were taken into consideration. Molecular global and atom-specific descriptors (see Figure S2) were extracted from Gaussian .log file or computed using the Python implementation Morfeus[21]. For each descriptor, ensemble-based values were collected, including the minimum (min), maximum (max), and the lowest-energy conformer (lowE) value, as well as the Boltzmann-weighted average (Boltz) values of the ensemble. In total, 164 descriptors were collected. The optimized XYZ coordinates and a complete list of descriptors are available in the project-related Git-Lab repository (https://git.rz.tu-bs.de/proppe-group/co2_affinity_prediction). When the carbon neighbors C_1 and C_2 were involved, averaged descriptors were generated, due to no clear distinction between the two carbon atoms.

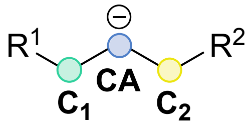

Figure S2: Atoms in nucleophile used for parameter acquisition. CA is the carbanionic site, C_1 and C_2 are the two carbon neighbors.

Global descriptors:

- HOMO and LUMO energies,
- Chemical potential, electrophilicity index, hardness,
- Polarizability,
- Dipole moment,
- Molecular volume,
- Solvent accessible surface area (SASA)

Atom specific descriptors for the nucleophiles:

- CA-C_1 and CA-C_2 distances (averaged),
- C_1-CA-C_2 angle,
- NBO charge on CA, C_1 and C_2,
- Hirshfeld charge for CA,
- NMR shift for CA,
- Sterimol L, B1, B5 (CA-C_1, CA-C_2) values (Morfeus) (averaged),
- Buried sterimol L, B1, B5 (CA-C_1, CA-C_2) values within 5.5Å (Morfeus) (averaged),
- Pyramidalization CA (Morfeus),
- %VBur for CA from 2 to 4 Å steps (Morfeus)

Section S4: Statistical modeling

To train a multivariate linear regression (MLR) model, we utilized a dataset comprising 31 nucleophiles, each characterized by the specific pattern: a carbanionic site with two carbon neighbors, as visualized in Figure S2. Prior to model training, the collected descriptors were normalized and filtered. This process included removing descriptors with collinearity (R^2 > 0.60) and those providing redundant information.

The development of the MLR model was carried out using the scikit-learn library in Python[22] and in-house scripts. The dataset was split into training and test sets in a 70%/30% ratio, employing the $y_{equidist}$ algorithm to ensure the points evenly span the output variable. Forward stepwise linear regression, implemented in an in-house script (https://github.com/SigmanGroup/python-modeling.git), was applied to generate different models, which were subsequently evaluated by statistical metrics such as the coefficient of determination (R^2) and the mean absolute error (MAE, in kcal/mol) for both the training and test set. In each modeling iteration, the program first tests each provided descriptor by performing a regression with it. The best-performing descriptor is retained for the next iteration, and the next-best descriptor is then selected. This process is repeated until the predetermined number of iterations is reached, three in this work. The test set (30 % of the dataset, including 9 reactions) achieved a high accuracy of R^2 = 0.95 and an MAE of 1.00 kcal/mol. To assess the consistency and overall robustness of the MLR model, we employed cross-validation techniques, including leave-one-out (LOO) analysis and k-fold cross validation (k=5) on the entire dataset. The averaged cross-validation score of 0.97, along with a LOO score of 0.97, indicates very good and stable model performance. The performance of all models including the descriptors applied in this work can be found in the project-related Git-Lab repository in the notebook https://git.rz.tu-bs.de/proppe-group/co2_affinity_prediction/-/blob/main/MLR_model/Mattlab_modelling_CO2A_model.ipynb .

Section S5: Benchmarking quantum chemical protocol

The computational settings required for the target calculation necessary for training the model were B3LYP-D3(BJ)/def2-TZVPD. To reduce overall computational time, cheaper methods were tested for the descriptor calculations. To assess whether this setting is needed for optimizing nucleophiles and extracting their properties, the training process was repeated. This time, properties derived from different quantum chemical method, including semi-empirical methods and generalized gradient approximation (GGA) functionals were used. In this process, the three parameters included in the final model remained fixed, while only the coefficients were reoptimized. Table S1 summarizes the resulting statistical metrics from these different tests.

Table S1: Statistical metrics for MLR model trained on properties obtained from quantum chemically optimized structures using different settings.

**Method**	**Basis set**	**R^2 training**	**MAE (kcal/mol) training**	**R^2 test**	**MAE (kcal/mol) test**	**LOO R^2**	**5-fold R^2**
B3LYP	def2-TZVPD	0.98	0.57	0.95	1.01	0.96	0.96
B3LYP	def2-SVPD	0.97	0.74	0.92	1.17	0.94	0.59
PBE	def2-TZVPD	0.98	0.55	0.95	1.07	0.97	0.97
PM6	-	0.73	2.12	0.65	2.79	0.61	0.59

The statistical metrics for the PBE-D3(BJ)/def2-TZVPD settings are very similar compared to those of the original B3LYP-D3(BJ)/def2-TZVPD settings. This leads us to conclude that property calculations can be obtained using PBE as the exchange–correlation functional instead of B3LYP. To compare the two settings, Figures S3 and S4 show the MLR models for both settings and the visualization of model parameters, respectively (see main text Figure 2A for more details). This leads to an overall reduction in computational cost by a factor of about 4 compared to the cost required to calculate CO_2 affinity using the B3LYP-D3(BJ)/def2-TZVPD method. No adduct calculations are required, which means that no conformer space needs to be sampled, and no structure optimizations and frequency calculations are

necessary

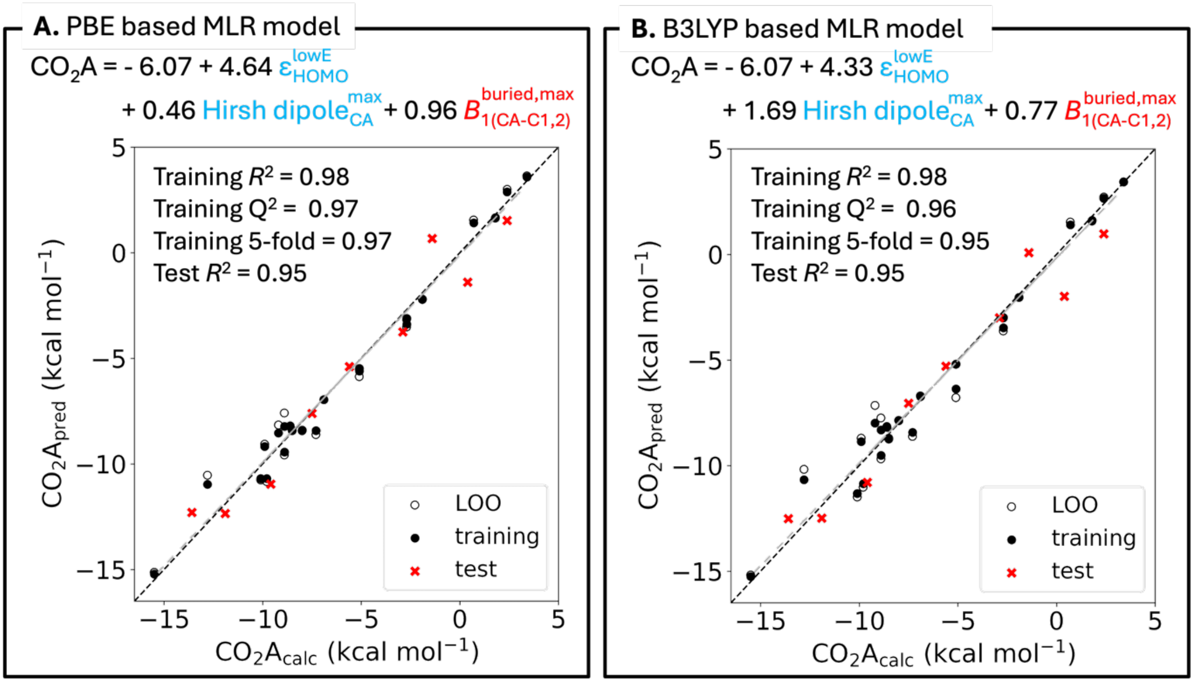

Figure S3: MLR model comparison for properties obtained from PBE-optimized nucleophiles in A and B3LYP-optimized nucleophiles in B.

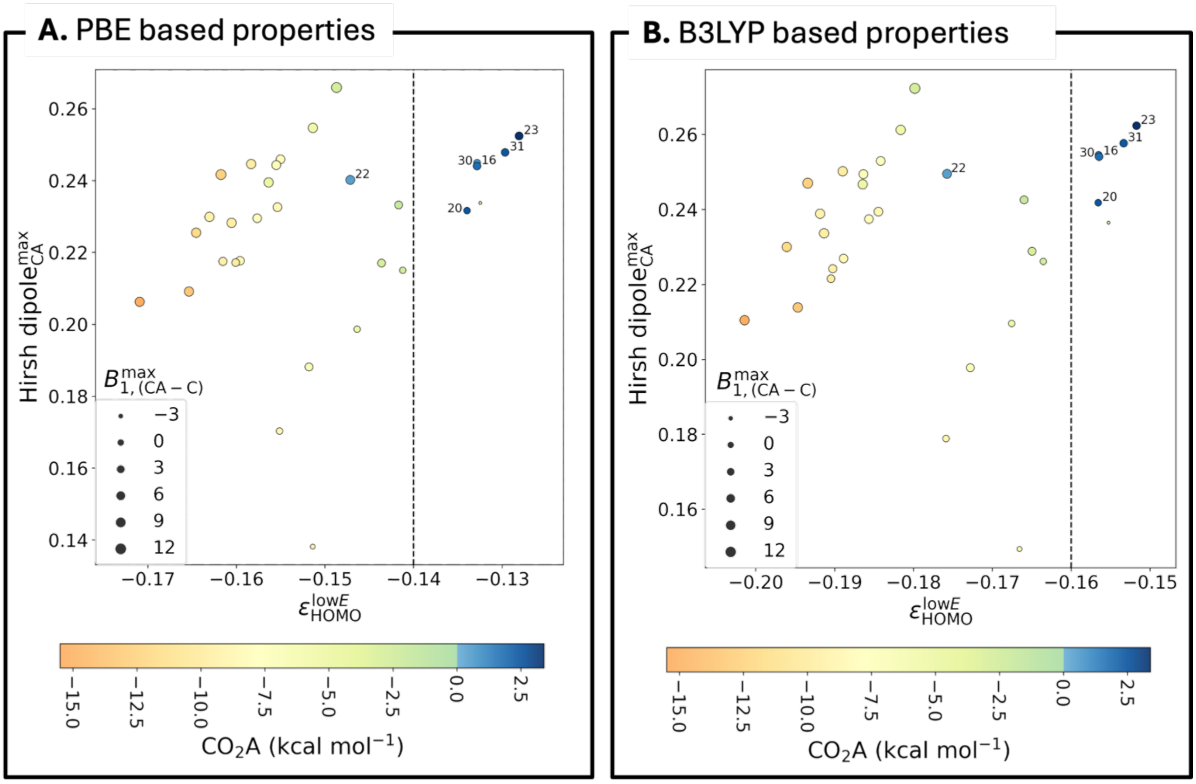

Figure S4: Visualization of properties included in the final MLR model in comparison for properties obtained from PBE-optimized nucleophiles in A and B3LYP-optimized nucleophiles in B.

Section S6: Computational model validation

To further validate the model, we employed quantum chemical methods to calculate the CO_2 affinities of the reactions included in the screening dataset. Only the reactions below the threshold ε_{HOMO} = −0.160 E_h

(for B3LYP settings) were included in the validation resulting in 39 reactions (left out: **64**-**67**, **75**-**80**, **83**-**85**, **89**; the calculations for **61**-**63**, **67**-**69**, **81**, **82** did not converge). Figure S5 shows a comparison between the calculated values and the predicted values from all reactions included in this study. While the model does slightly overestimate the CO_2 affinities in the screening dataset (blue crosses), the overall accuracy is still very good with R^2 = 0.94. This approach ensures that no important nucleophiles are missed.

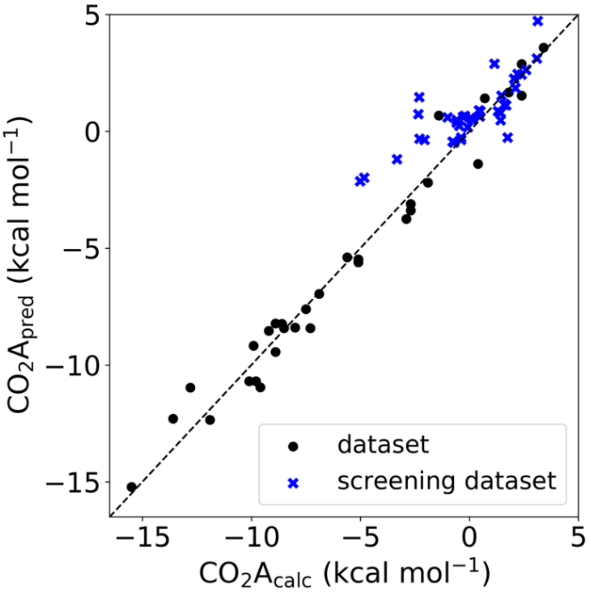

Figure S5: Comparison between the calculated CO_2 affinities and the predicted CO_2 affinities from all reactions included in the initial dataset (black circles) and 39 reactions of the screening dataset (blue crosses) with R^2 = 0.94.

Observed relationship with experimental yield - The CO_2 affinity (CO_2A, kcal·mol^{-1}) represents the computed thermodynamic driving force for CO_2 binding to the nucleophile. Across the experimental set studied, nucleophiles with $CO_2A \approx +2$ to +4 kcal·mol^{-1} typically afford moderate yields (≈30–40%) in toluene, consistent with a favorable but reversible formation of the CO_2 adduct. In contrast, strongly negative CO_2A values (–2 to –6 kcal·mol^{-1}) correlate with low or negligible reactivity. While CO_2A provides a qualitative guide, kinetic and solvent effects (e.g., stabilization in DMSO) also influence experimental yields.

Section S7: Experimental validation of the predicted CO_2 adduct formation with different substrates and consecutive protonation

Reactivity in DMSO

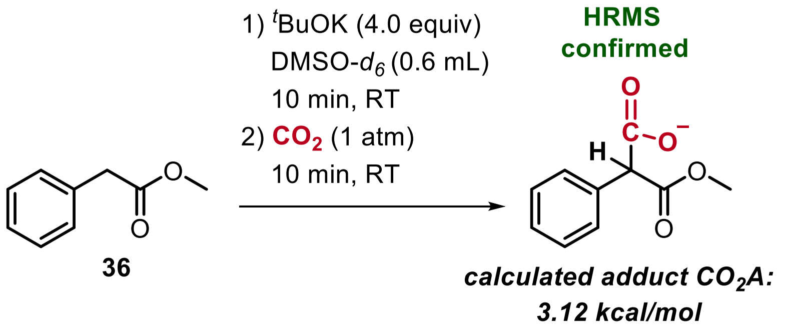

General procedure: Inside the glovebox, KO^{t}Bu (75 mg, 0.67 mmol, 4.0 equiv.) was weighted into a Young NMR tube followed with the addition of DMSO-d_6 (0.6 mL) (CAS No = 175943, Sigma Aldrich) and the substrate (0.167 mmol, 1.0 equiv.). After this step, the gas cylinder was attached to the J. Young NMR tube and the CO_2 was passed at a pressure of 1 atm. The crude mixture was then directly analyzed by HRMS direct injection method.

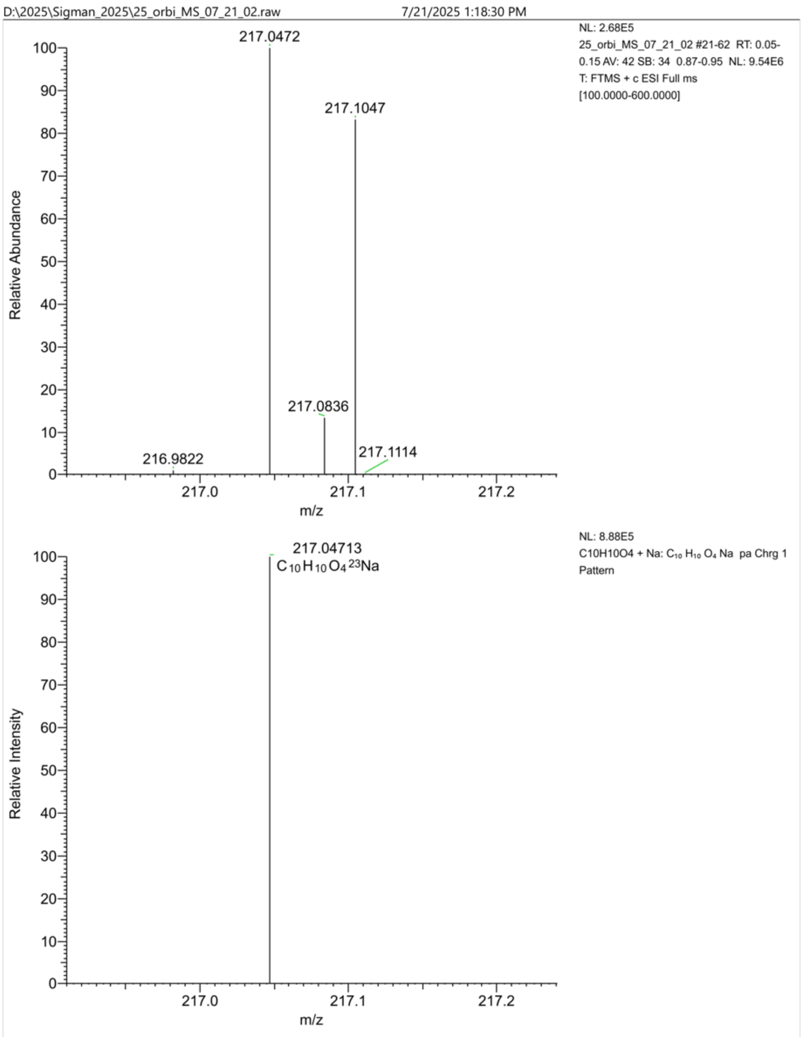

Figure S6: Experimental and predicted spectra of product **38-CO_2Na.**

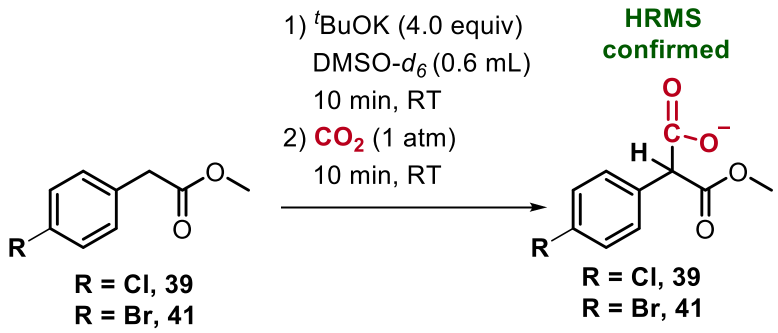

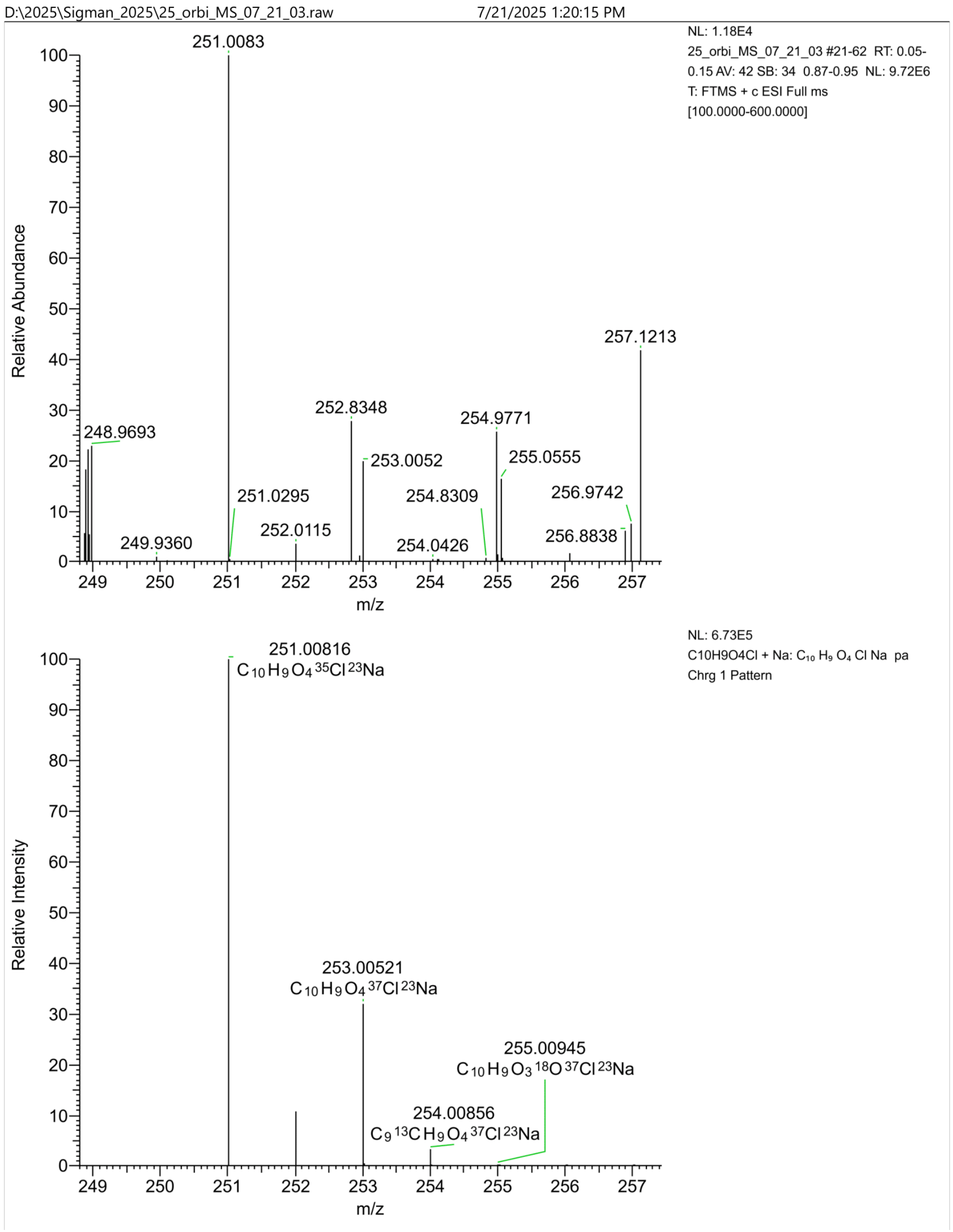

Figure S7: Experimental and predicted spectra of product **39-CO_2Na.**

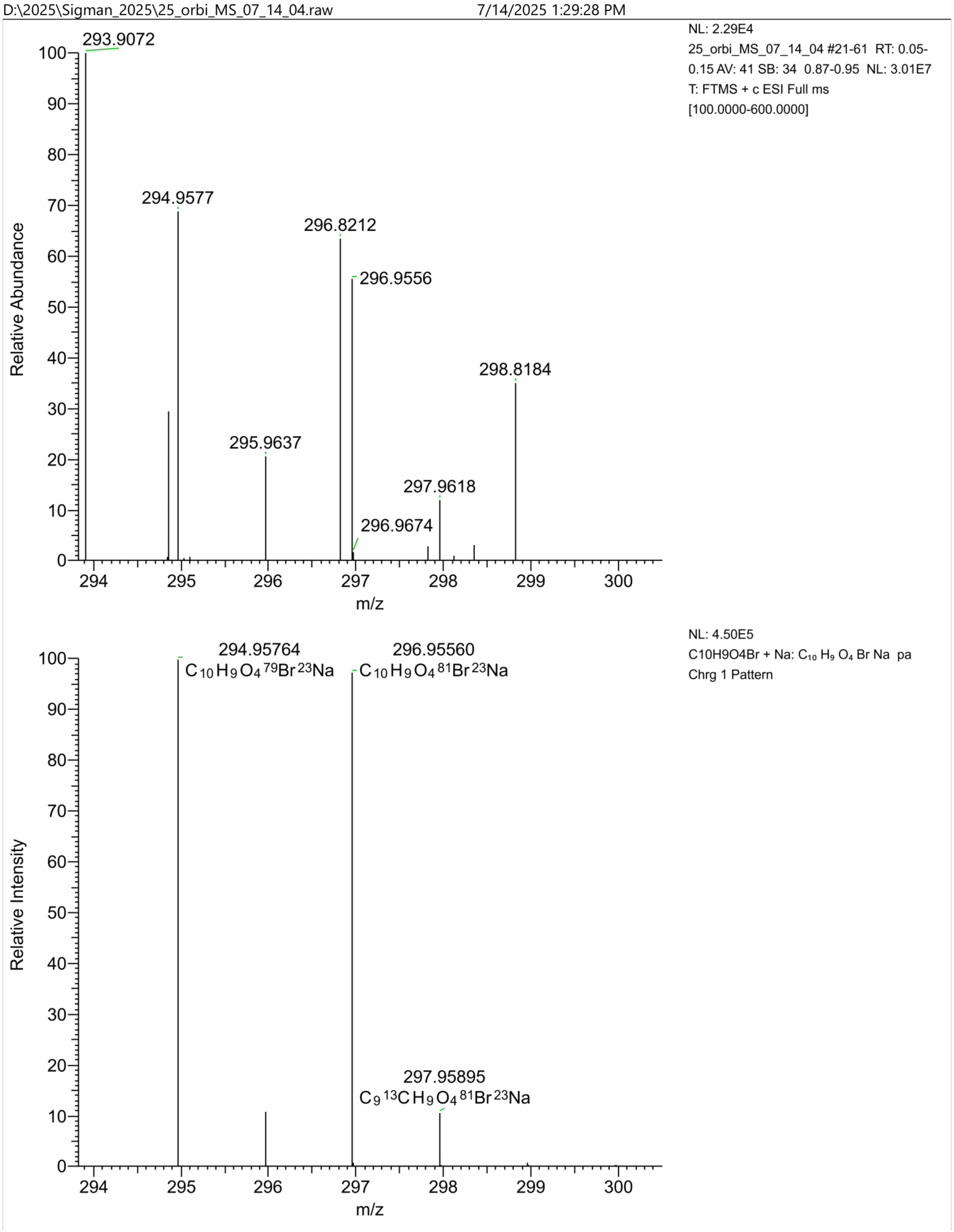

Figure S8: Experimental and predicted spectra of product **41-CO_2Na.**

Reactivity in Toluene

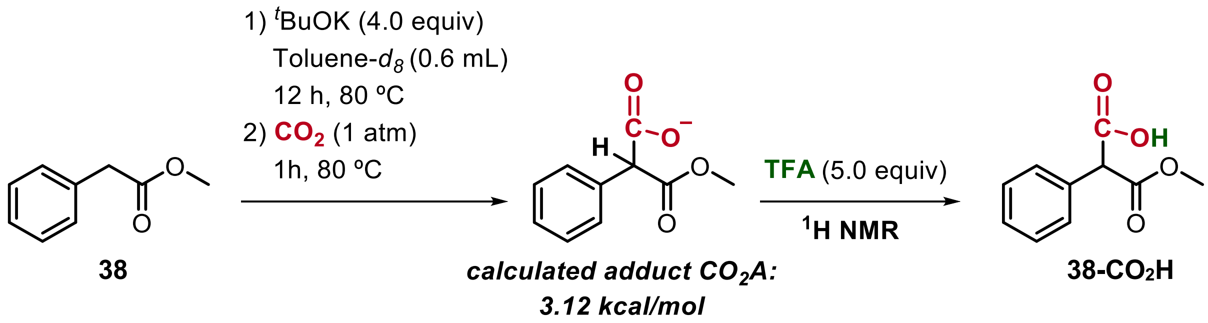

Scheme S1: Reaction scheme of methyl 2-phenylacetate carboxylation with CO_2 and TFA.

General procedure: Inside the glovebox, KO^{t}Bu (75 mg, 0.67 mmol, 4.0 equiv.) was weighted into a Young NMR tube followed with the addition of Toluene-d_8 (0.6 mL) and the substrate (0.167 mmol, 1.0 equiv.). Outside the glovebox, the mixture was heated at 80 °C for 12h. After this step, the gas cylinder was attached to the Young tube and the **CO_2** was passed at a pressure of 1 atm. The mixture was heated for 1h more at 80 °C and then **TFA** acid (64 µL, 0.83 mmol, 5.0 equiv.) and 1,3,5-trimethoxybenzene (28 mg, 0.167 mmol, 1.0 equiv.) -internal standard- were added before the sample was measured after 1h by ^{1}H NMR for yield quantification. Later, the same sample was used to verify the product formation by HRMS method.
Note: The yields were measured by ^{1}H NMR with a relaxation delay of 1.5 seconds, which is sufficient for semi-quantitative integration in our case due to the excellent signal-to-noise ratio and the use of a well-resolved singlet with minimal overlap."

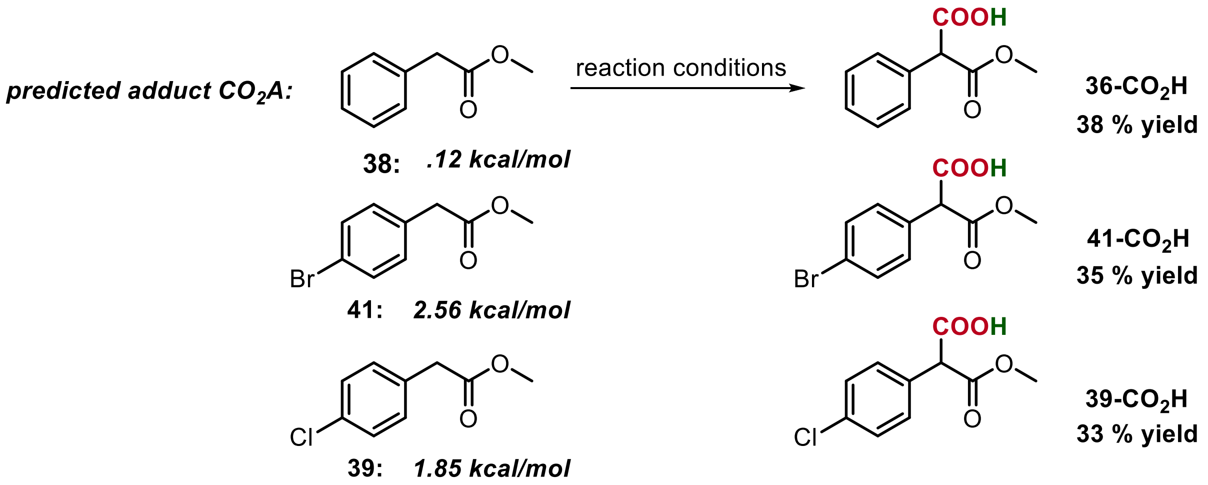

Scheme S9: Reported NMR yield of the carboxylation reaction with different predicted substrates.

<u>^{1}H NMR spectra of the protonated predicted substrates:</u>

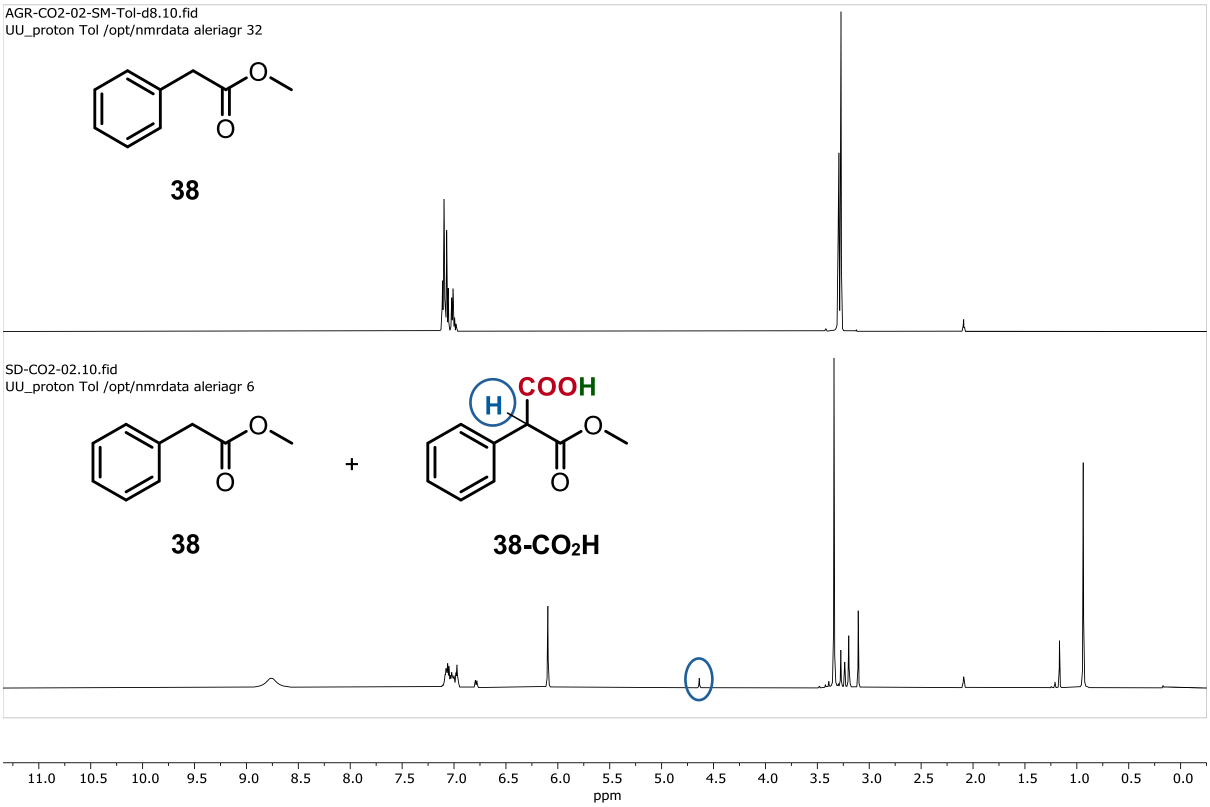

Figure S10: ^{1}H NMR spectra of product **38-CO$_2$H** in the reaction crude compared to the starting material **38**.

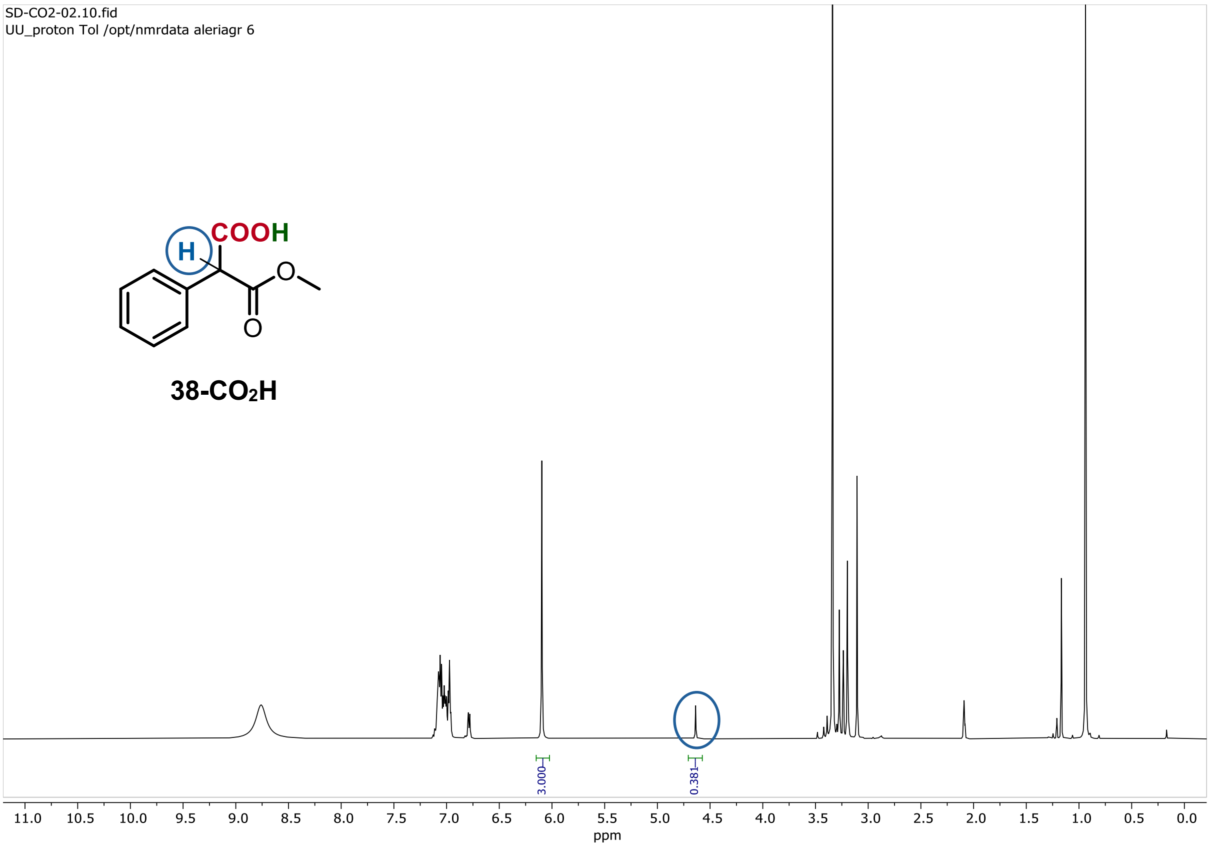

Figure S11: ^{1}H NMR spectra of product **38-CO₂H** in the reaction crude with a 38% yield. Signals matched with the previously reported spectra in the literature.

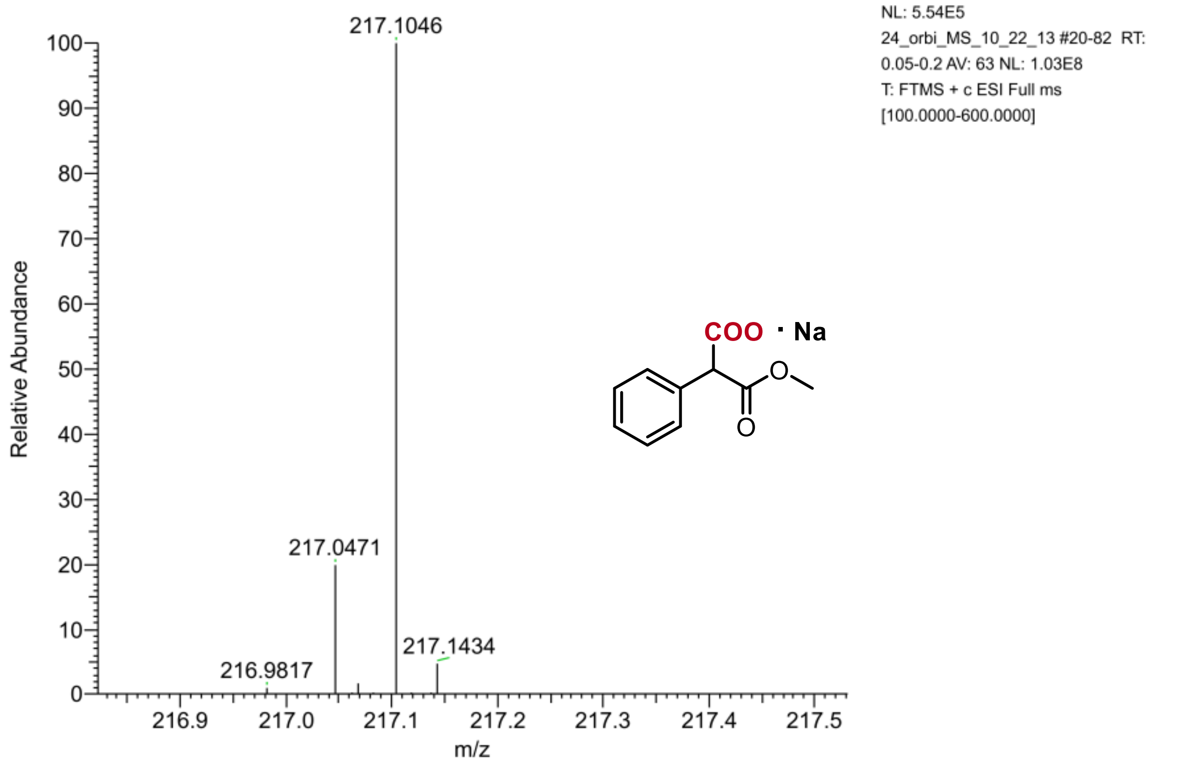

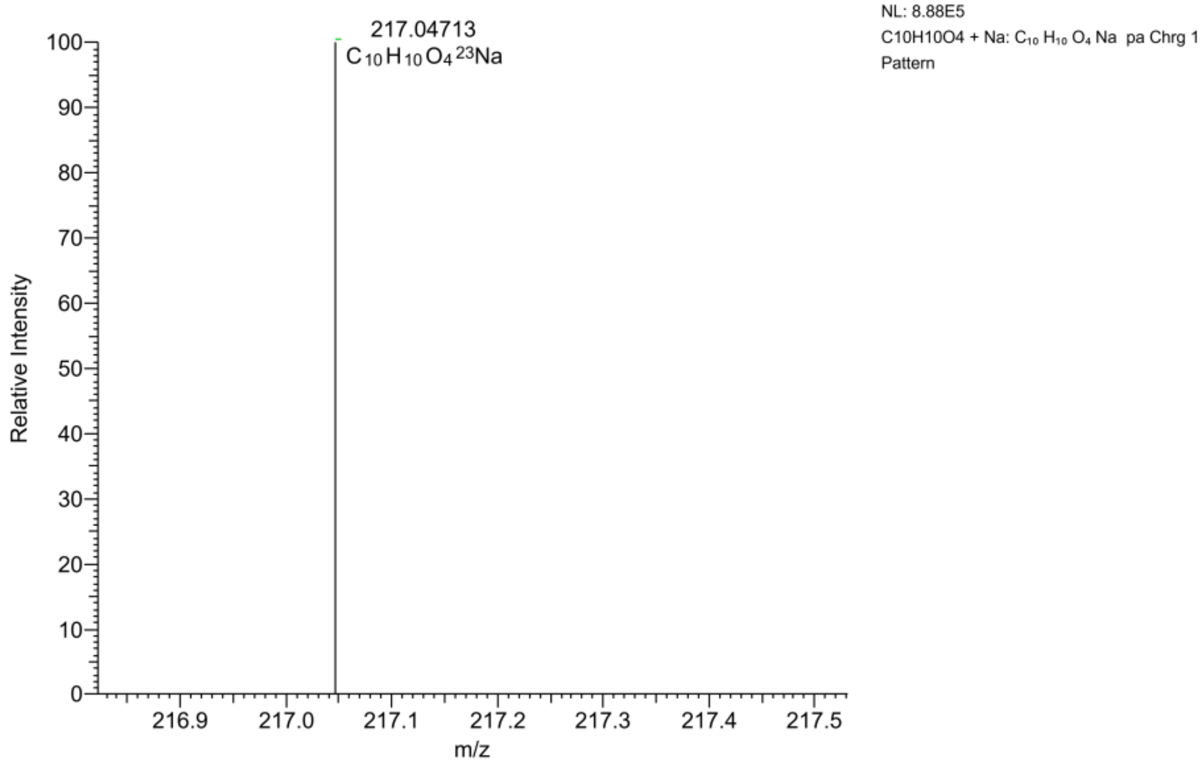

Figure S12: Experimental and predicted spectra of product **38-CO_2Na**.

In addition to the expected [M + Na]$^+$ ion of the desired compound ($C_{10}H_{10}O_4$Na, m/z 217.0472), an additional peak at m/z 217.1047 was detected. The large mass deviation (≈ 0.0575 Da, ~275 ppm) confirms that this signal does not correspond to the same molecular species. This peak likely originates from a minor fragment or adduct generated under the electrospray ionization conditions, possibly involving loss or exchange of small neutral species. As its intensity is comparable to that of the molecular ion, we acknowledge that it may represent a related oxygenated species rather than a random impurity. No evidence for this ion was observed in NMR analysis, suggesting that it arises during ionization rather than being present in bulk solution."

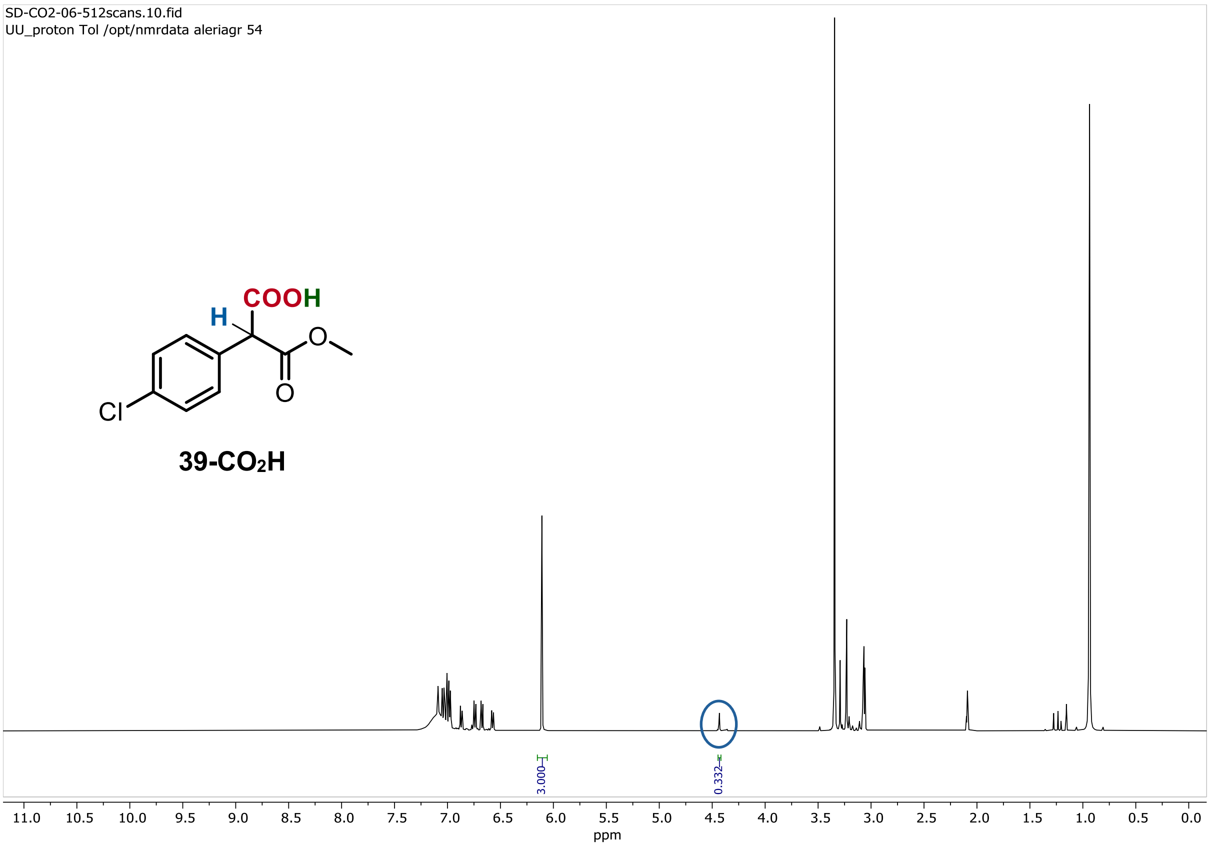

Figure S13: ^{1}H NMR spectra of product **39-CO_2H** in the reaction crude with a 33% yield. Signals matched with the previously reported spectra in the literature.

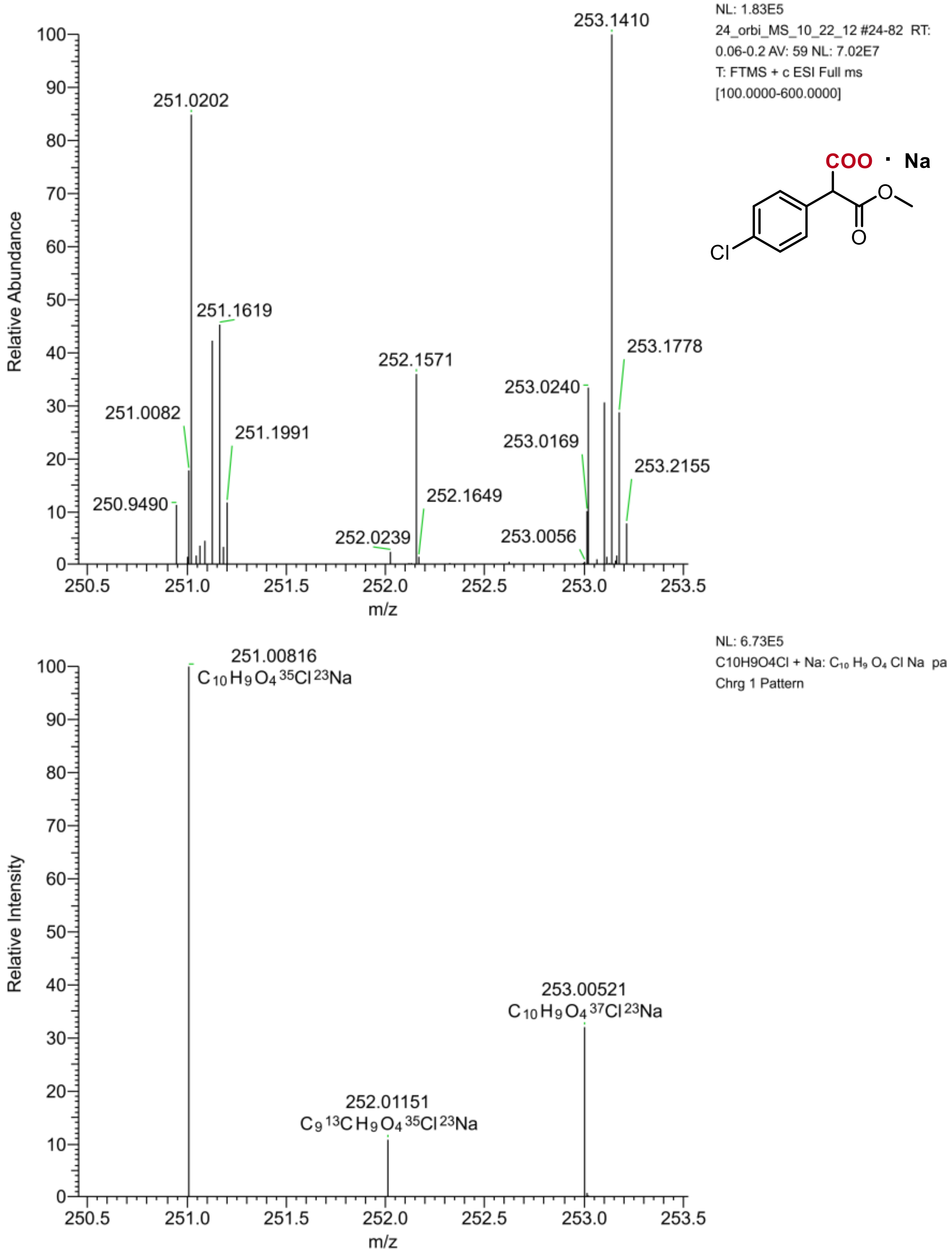

Figure S14: Experimental and predicted spectra of product **39-CO_2Na**.

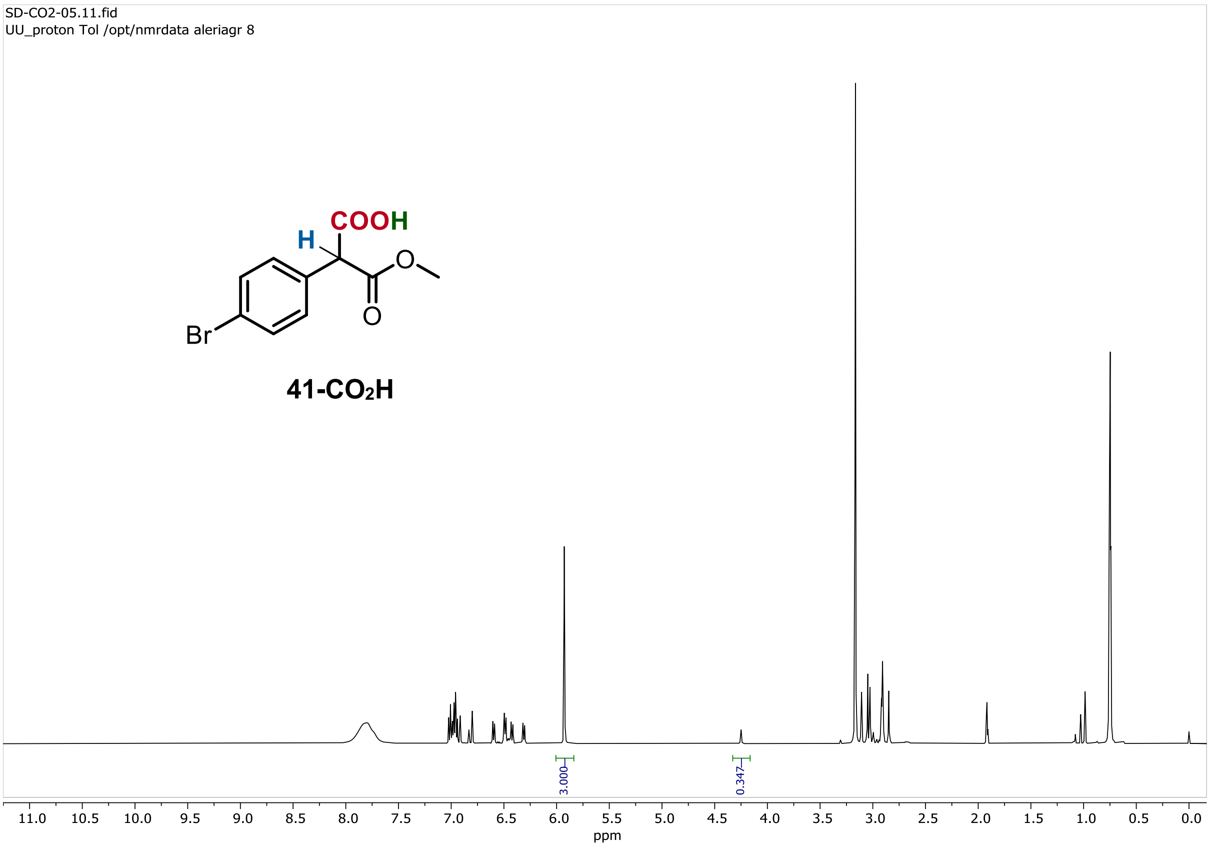

Figure S15: ^{1}H NMR spectra of product **41-CO₂H** in the reaction crude with a 35% yield.

Isolated spectrum

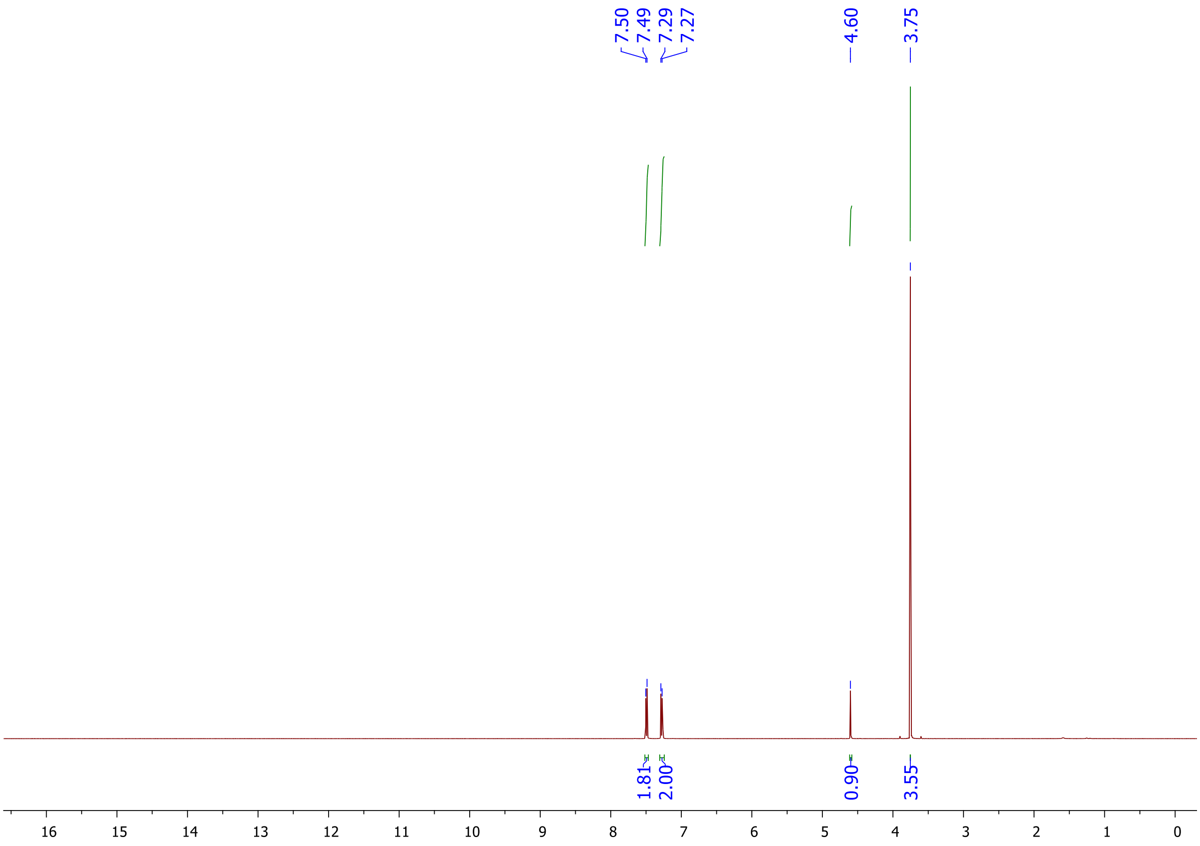

Figure S16: ^{1}H NMR spectra of product **41-CO₂H**.

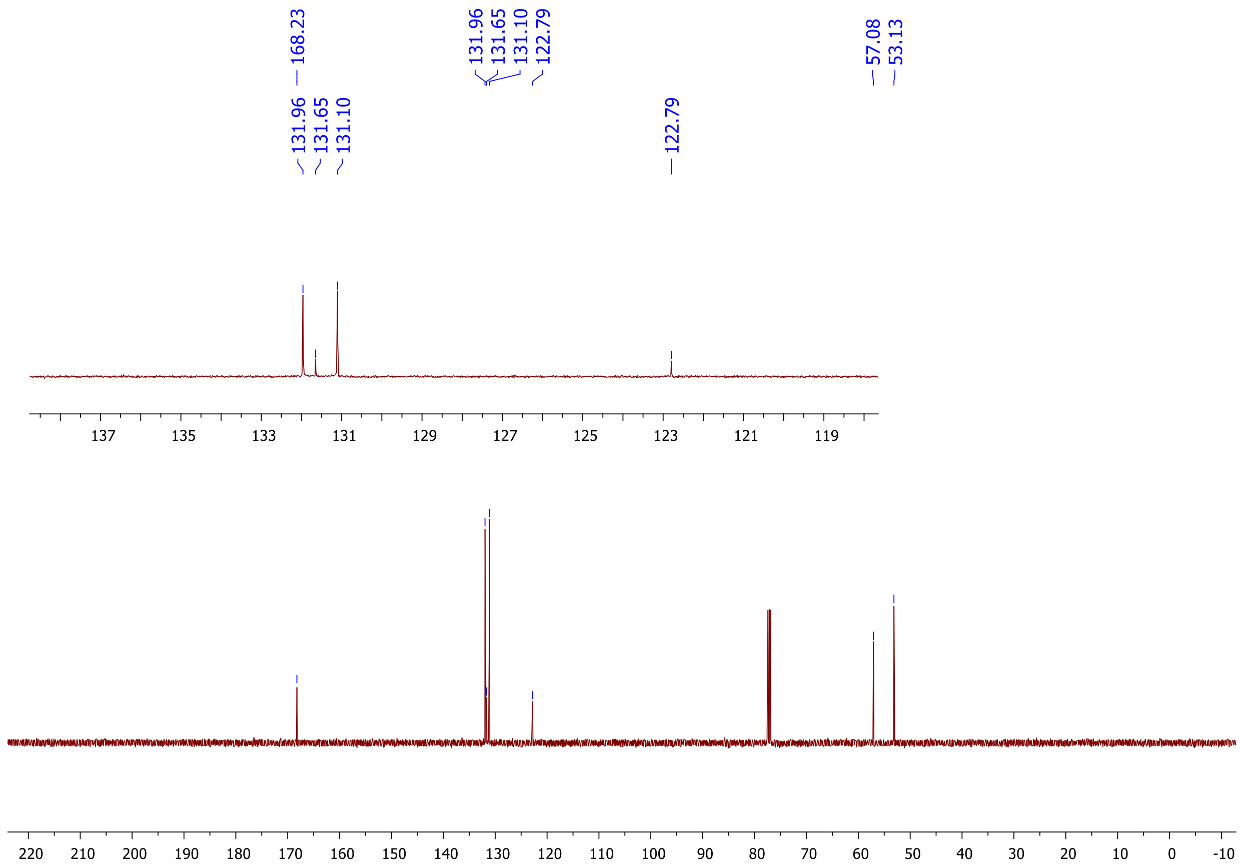

Figure S17: ^{13}C NMR spectra of product **41-CO₂H**.

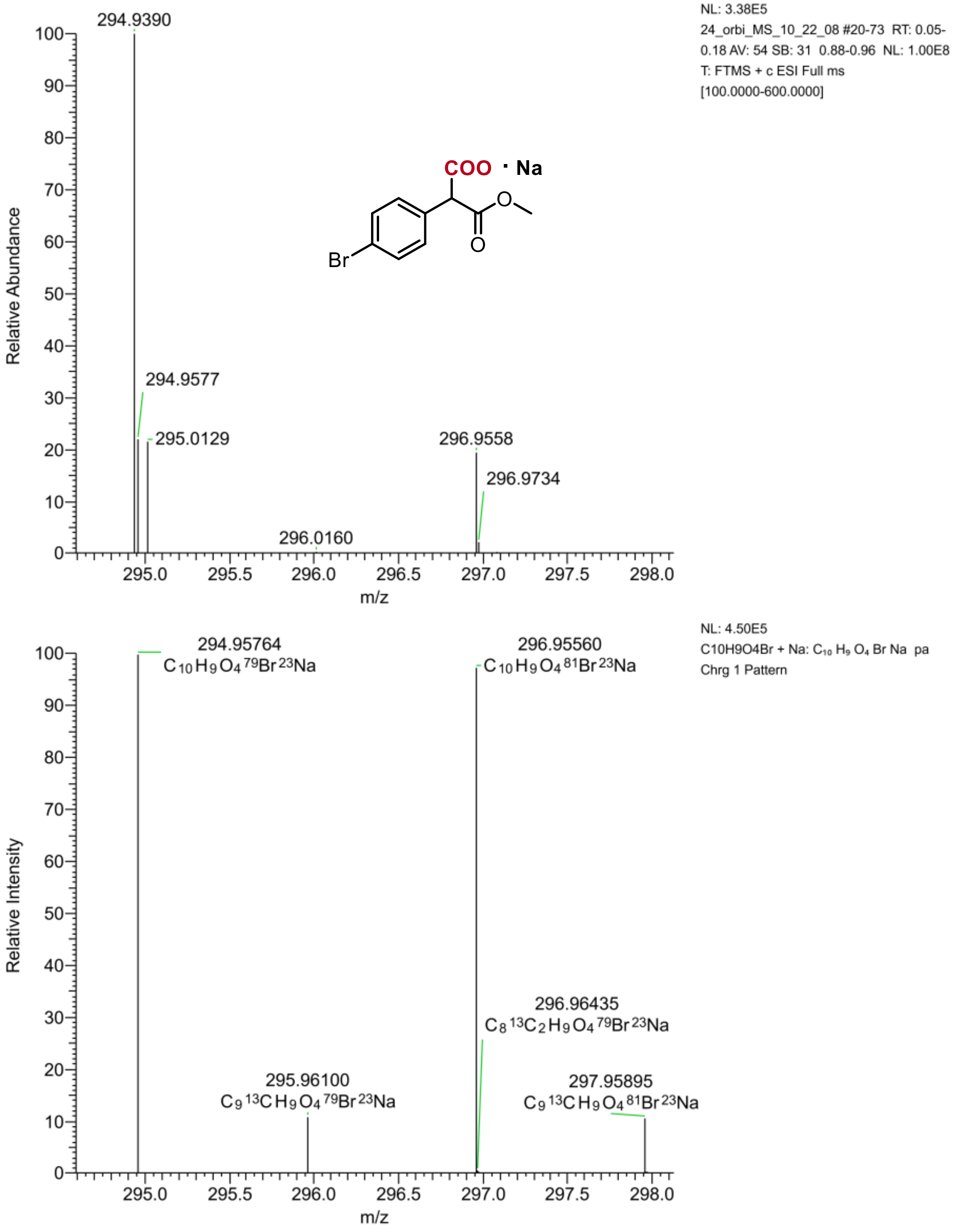

Figure S18: Experimental and predicted spectra of product 41-**CO_2Na**.

^{1}H NMR spectra of the non-reactive substrates:

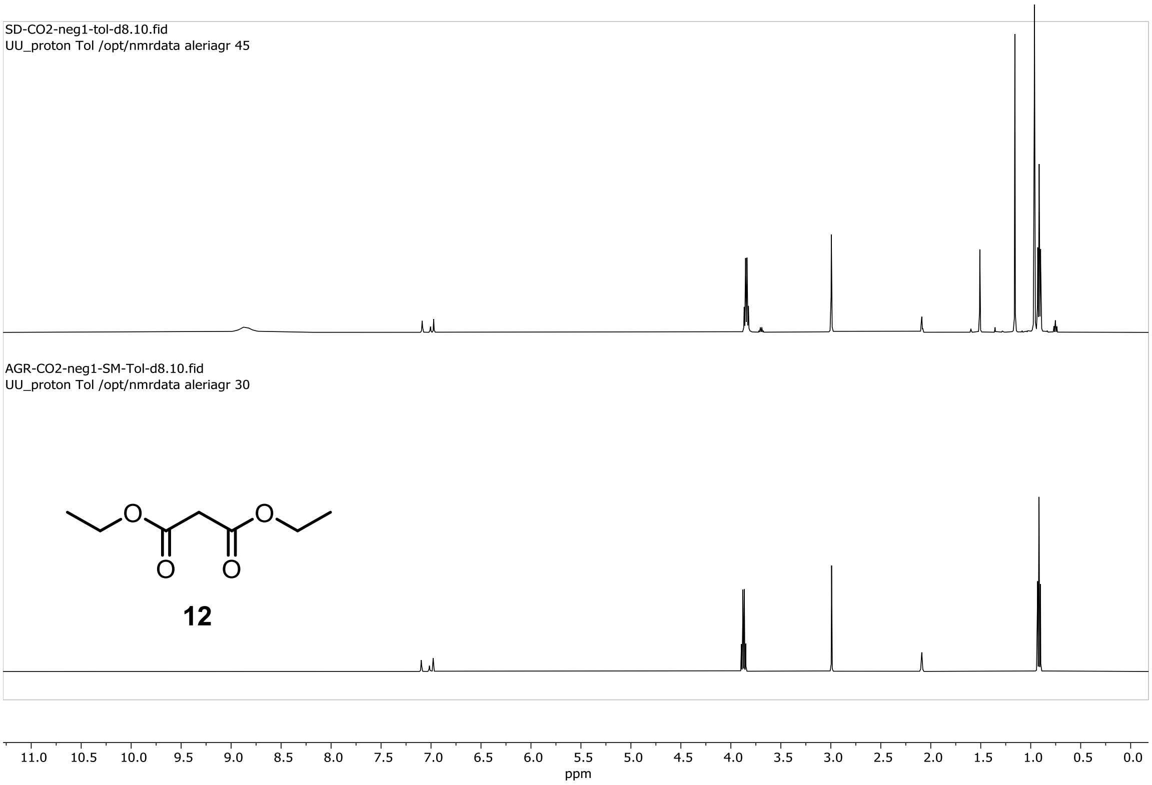

Figure S19: ^{1}H NMR spectra of almost unreacted substrate **12** in the reaction mixture with $^{t}BuOK$ and TFA (top) compared to the starting material (bottom). 2.6% conversion of the carboxylated product can be detected by ^{1}H NMR (see zoomed spectra).

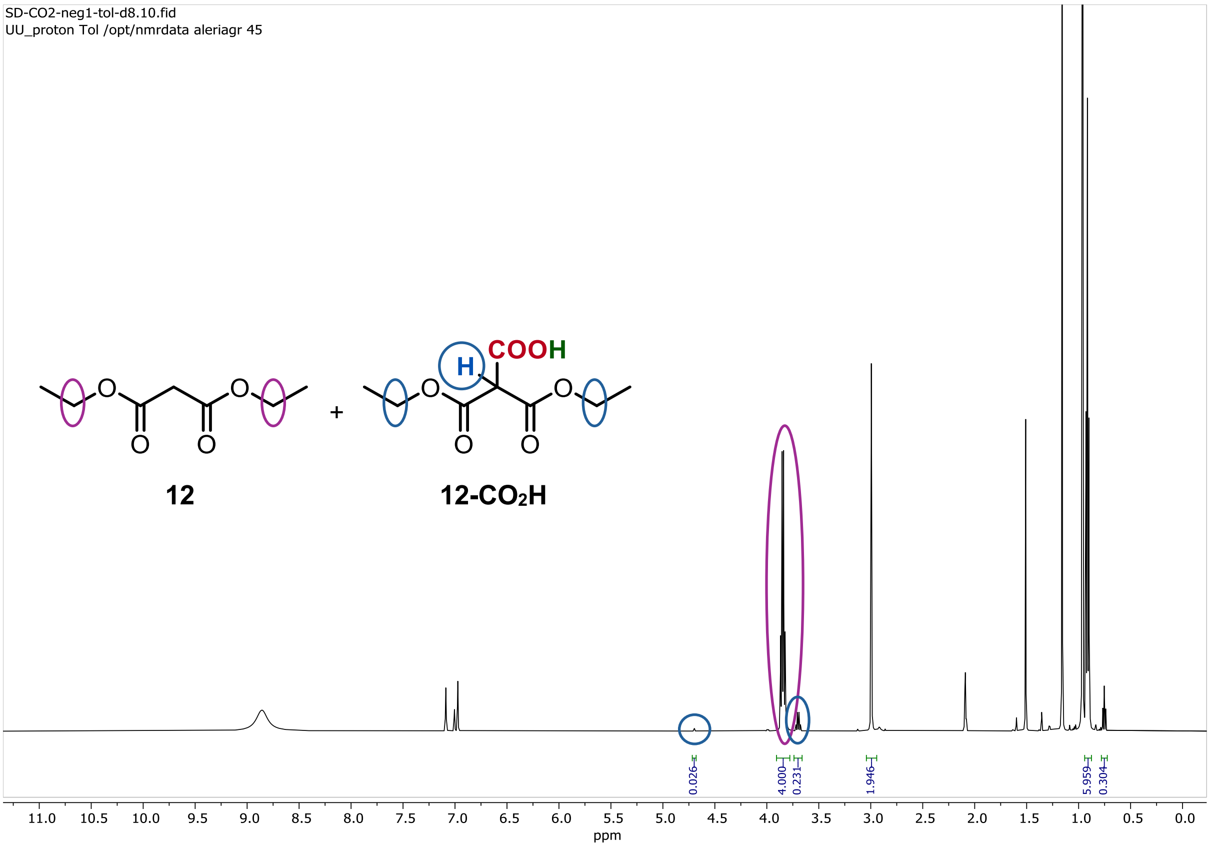

Figure S20: Zoomed ^{1}H NMR spectra of substrate **12** reaction crude where a 2.6% conversion towards product **12-CO_2H** can be detected.

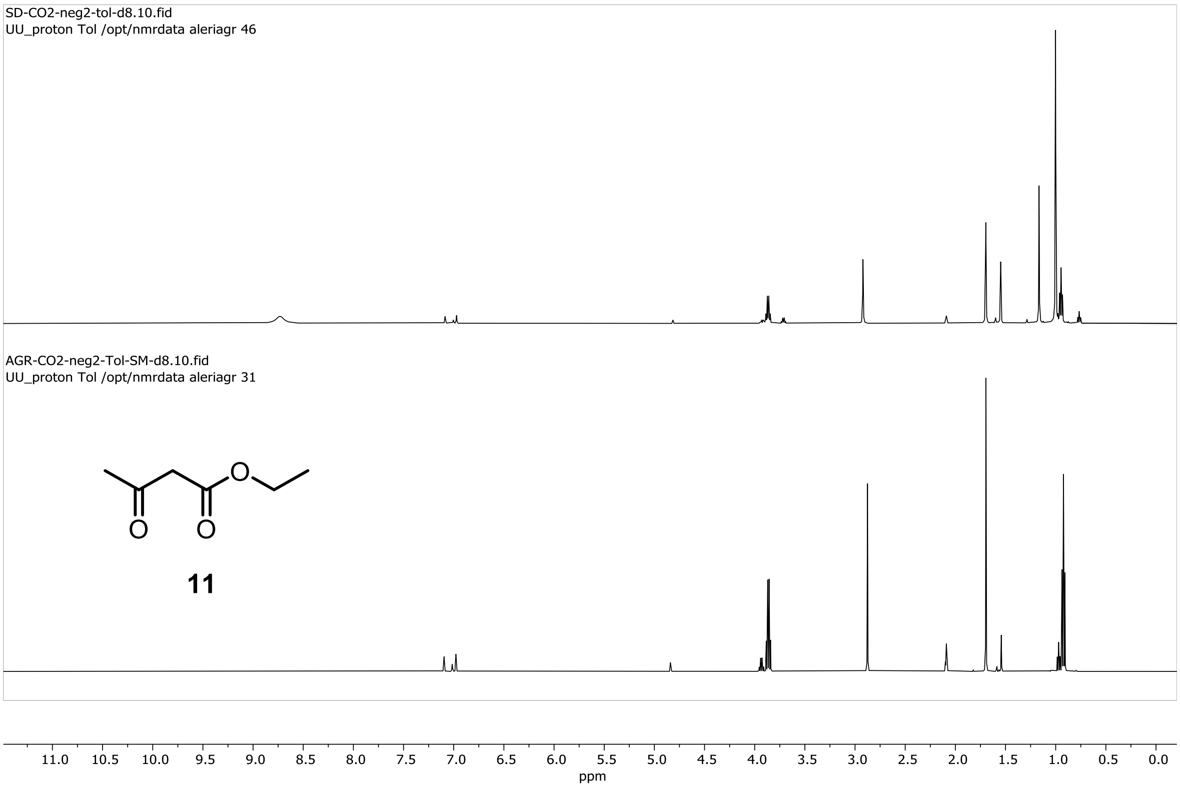

Figure S21: ^{1}H NMR spectra of almost unreacted substrate **11** in the reaction mixture with ^{t}BuOK and TFA (top) compared to the starting material (bottom). 5.9% conversion of the carboxylated product can be detected by ^{1}H NMR (see zoomed spectra).

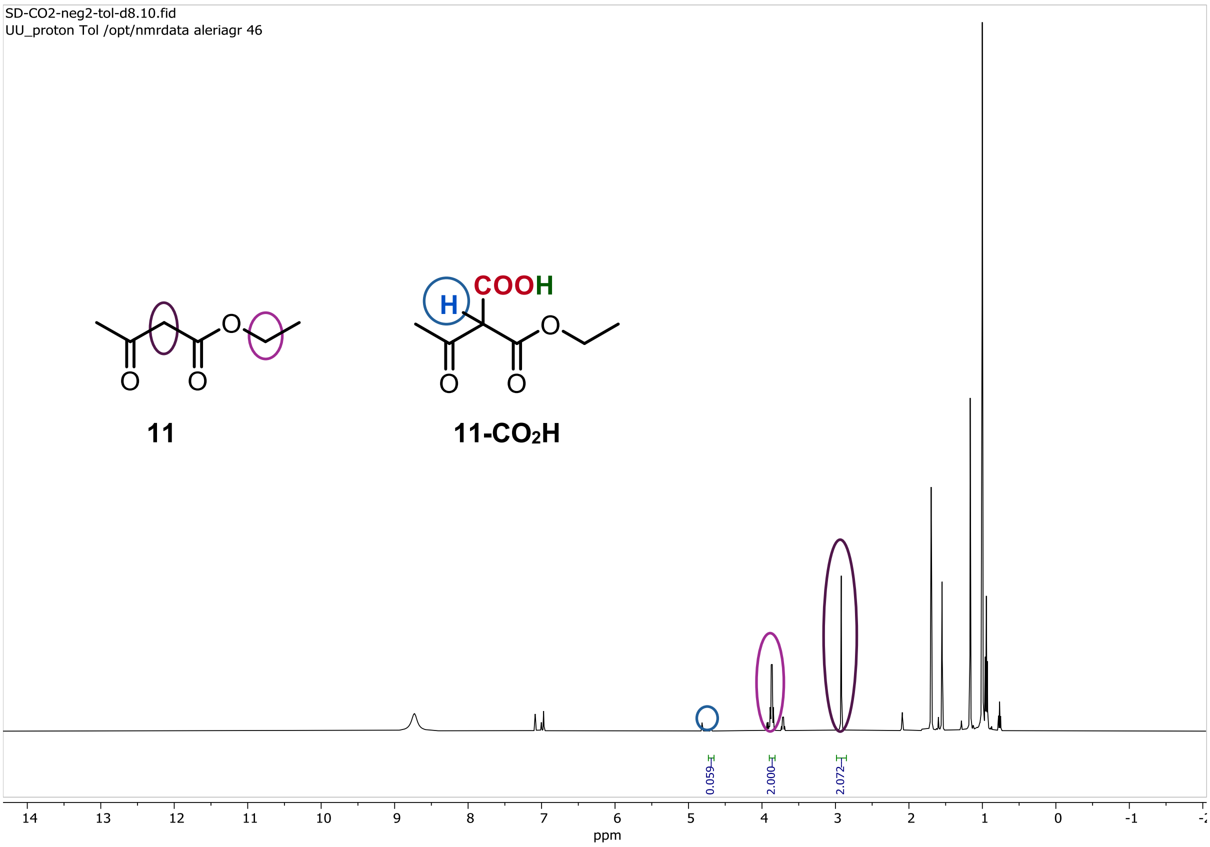

Figure S22: Zoomed ^{1}H NMR spectra of substrate **11** reaction crude where a 5.9% conversion towards product **11-CO$_2$H** can be detected.

Reactivity in DMSO

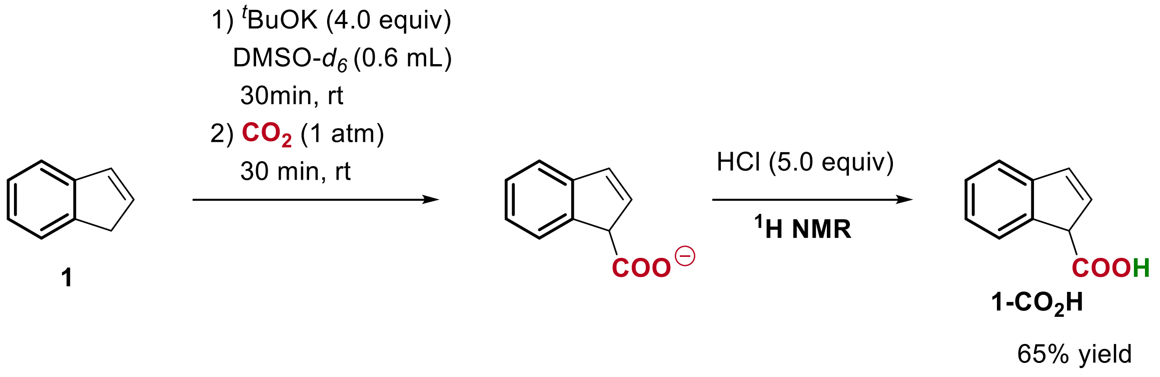

Scheme S3: Reaction scheme of indene carboxylation with CO_2 and TFA.

General procedure: Inside the glovebox, KO^{t}Bu (75 mg, 0.67 mmol, 4.0 equiv.) was weighted into a Young NMR tube followed with the addition of DMSO-d_6 (0.6 mL) and the substrate (0.167 mmol, 1.0 equiv.). Outside the glovebox, the mixture was spinned at room temperature for 30 min. After this step, the gas cylinder was attached to the Young tube and the CO_2 was passed at a pressure of 1 atm. The mixture was spinned for 30 min more at room temperature and then **HCl** acid (35% v/v) (0.83 mmol, 5.0 equiv.) and 1,3,5-trimethoxybenzene (28 mg, 0.167 mmol, 1.0 equiv.) -internal standard- were added before the sample was measured after 1h by ^{1}H NMR for yield quantification. Later, the same sample was used to verify the product formation by HRMS method. For isolation, the crude mixture was dissolved in 5mL of water and extracted with EtOAc (5mL x2) after the HCl quenching step.

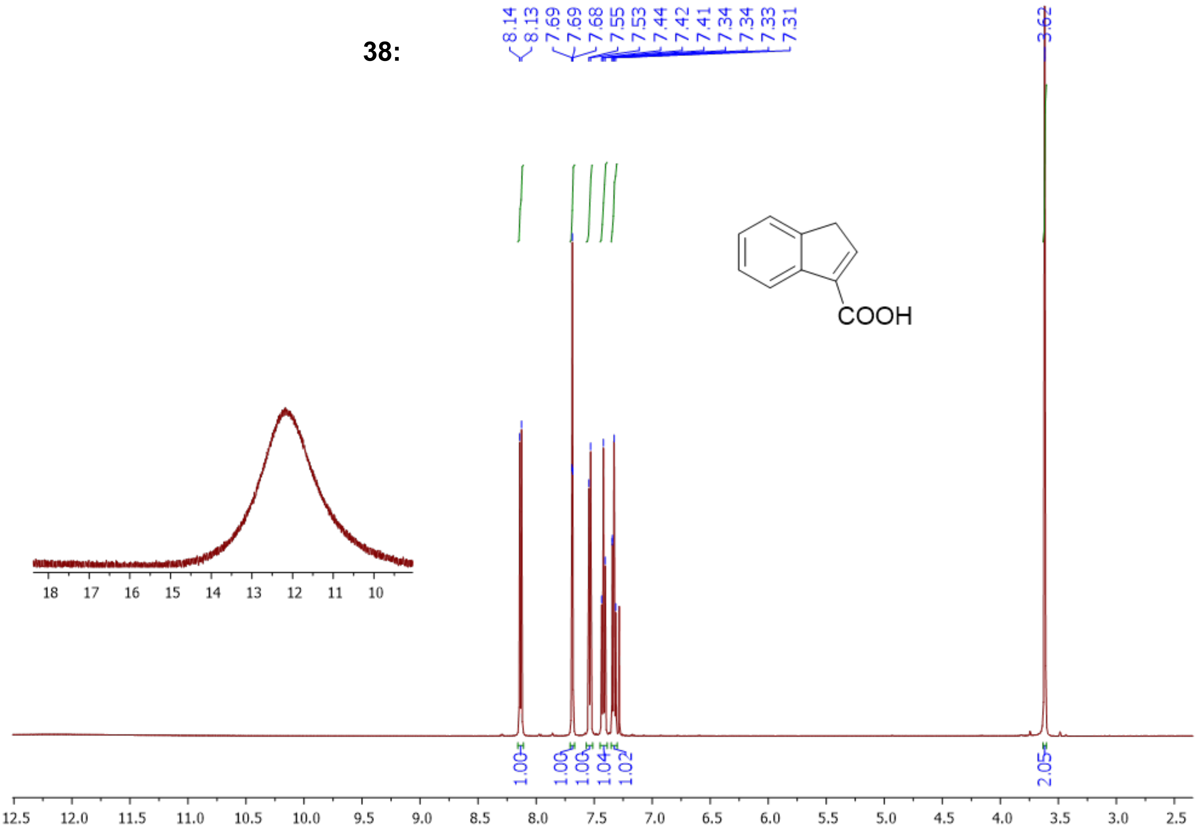

Figure S23: ^{1}H NMR spectra of isolated carboxylated indene; product 1-CO_2H.

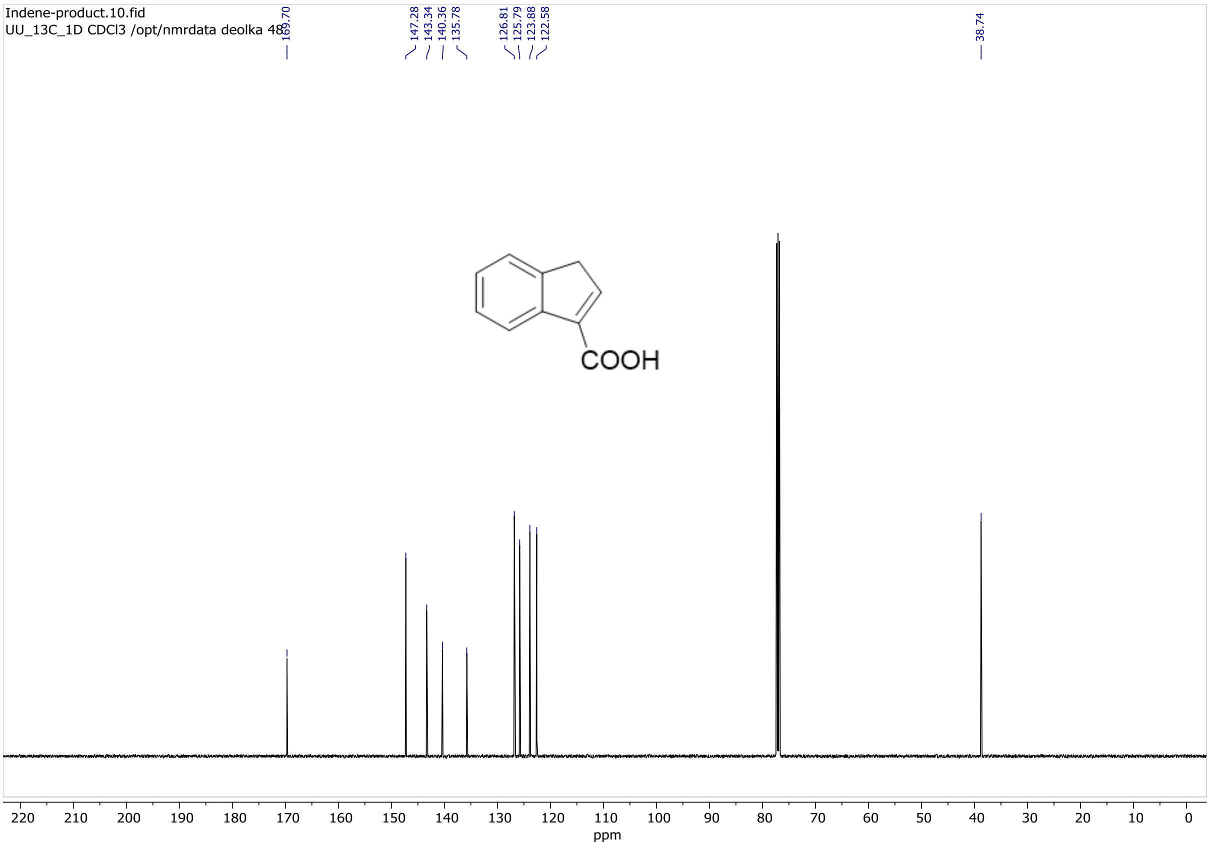

Figure S24: ^{13}C NMR spectra of isolated carboxylated indene; product 1-CO_2H.

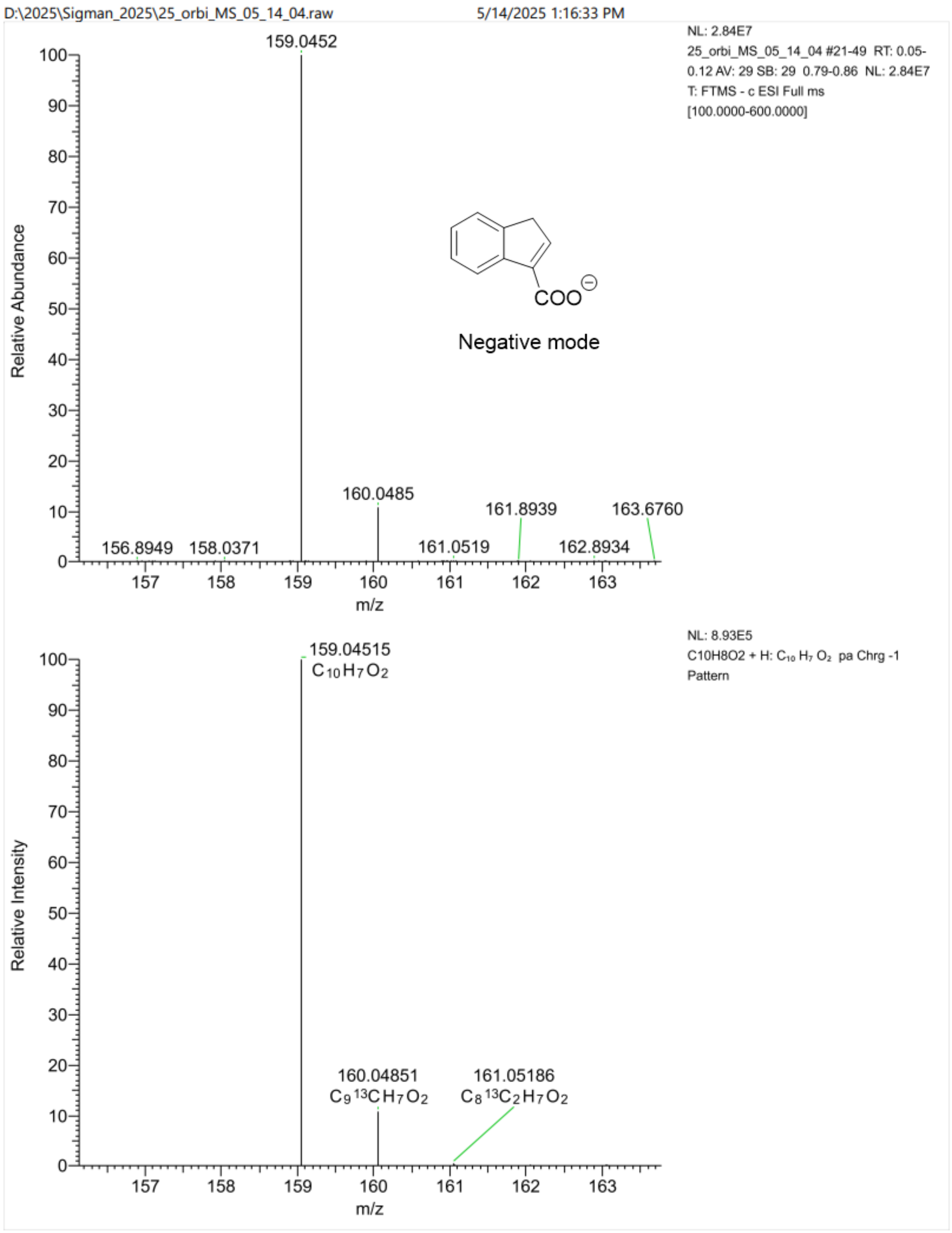

Figure S25: Experimental and predicted HRMS spectra of carboxylated indene; product **1-CO_2H**.

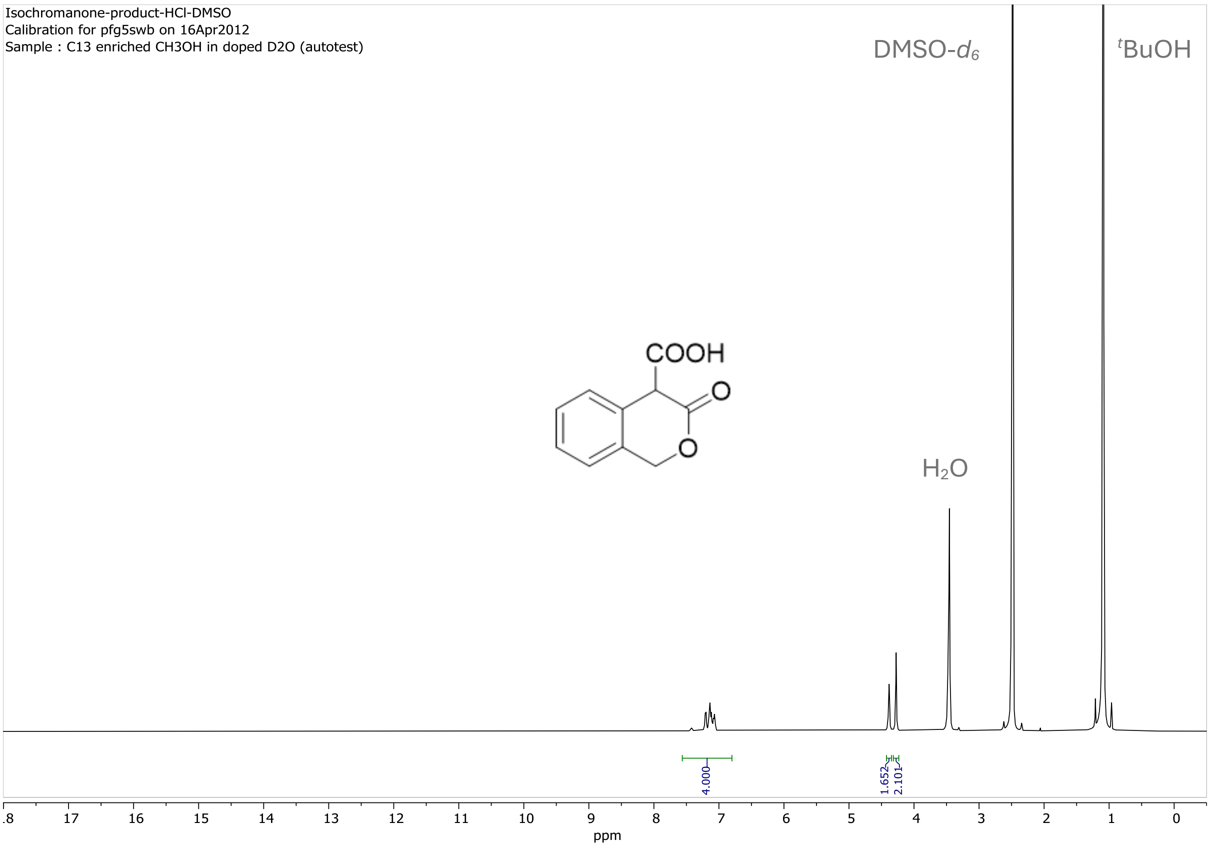

Figure S26: ^1H NMR spectra of isolated carboxylated isochromanone; product **92-CO₂H.**

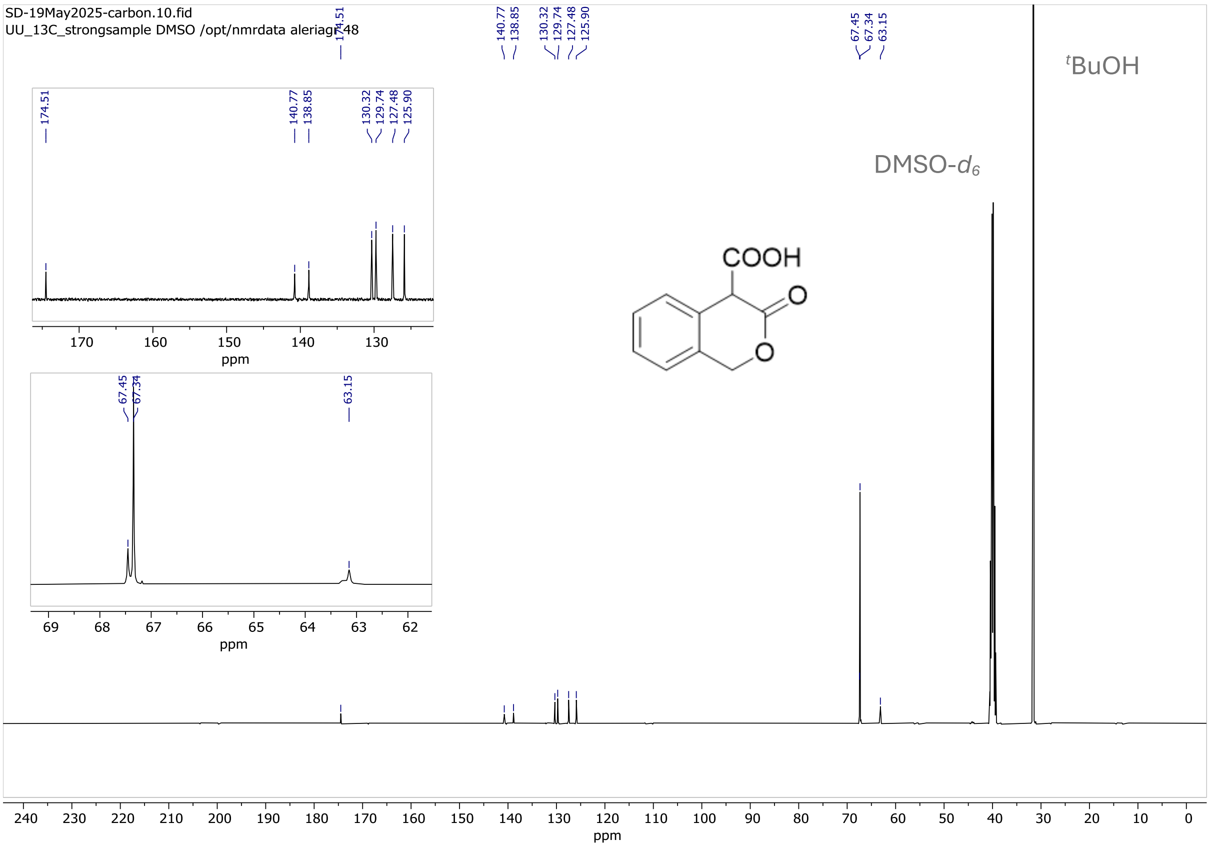

Figure S27: ^{13}C NMR spectra of isolated carboxylated isochromanone; product **92-CO$_2$H.**

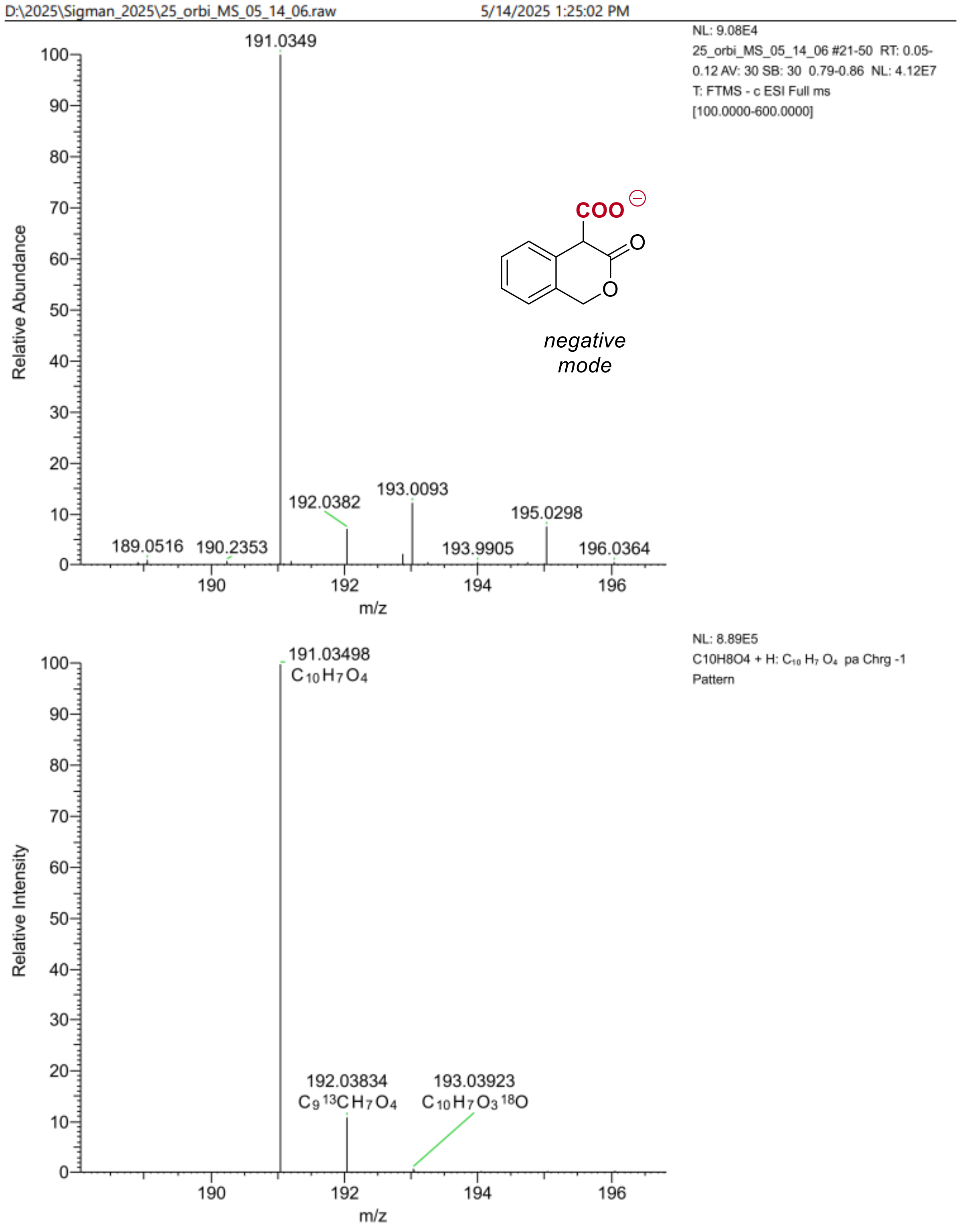

Figure S28: Experimental and predicted HRMS spectra of carboxylated isochromanone; product **92-CO_2H**.

Stability test in DMSO of isolated product

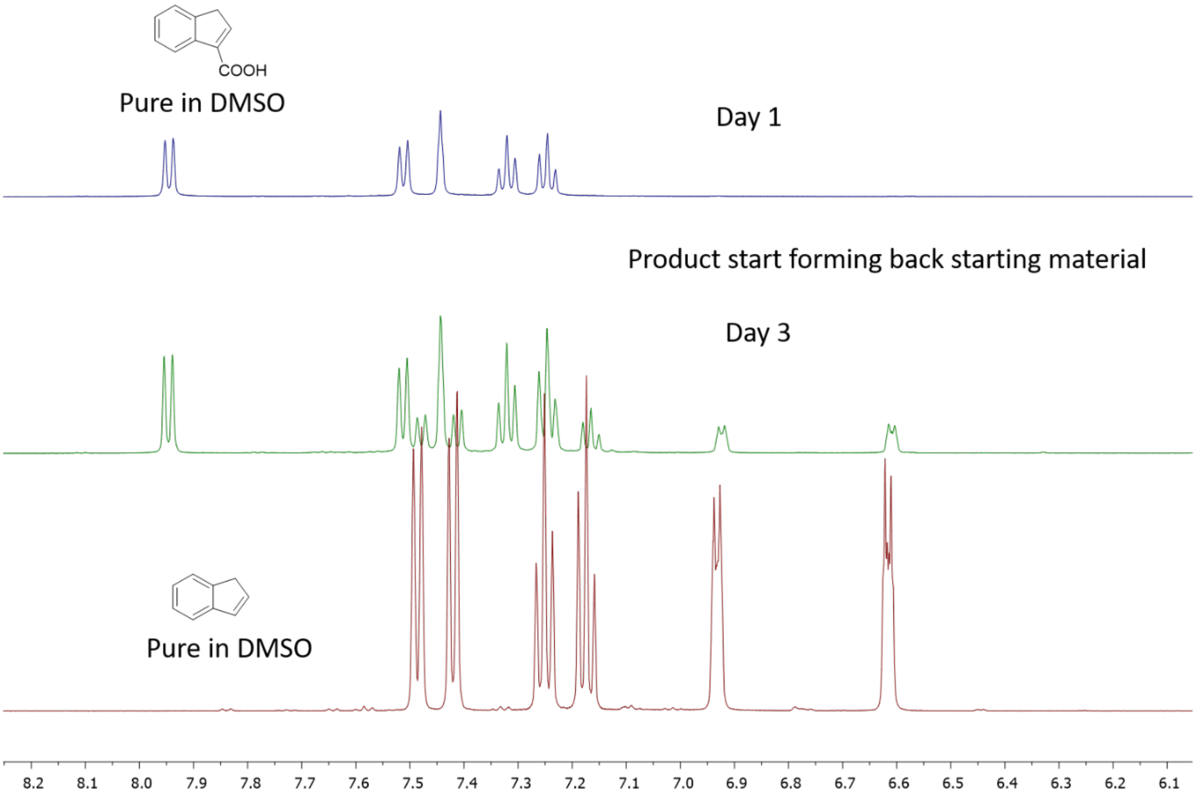

Figure S29: This experiment demonstrates the low thermodynamic stability of **1-COOH** product in DMSO.

Section S8: Correlation of nucleophilicity parameter *N* with CO_2 affinity

A correlation of the reactivity and stability can be useful to obtain information about the underlying reaction. In this study, we found a loose correlation between the reactivity, expressed as Mayr's nucleophilicity *N*[23], and the CO_2 affinity as stability measure with R^2 = 0.74 for dataset (31 reactions). We

can define a rule of thumb, that higher nucleophilicity corresponds to lower kinetic barriers and more stabilized products, leading to increased CO_2 affinity. In Figure S17, the correlation is visualized for the dataset with 31 reactions. If nucleophiles with unique patterns throughout the dataset are removed (i.e. **20**, **22**, **27**, **29**), R^2 improves to 0.86.

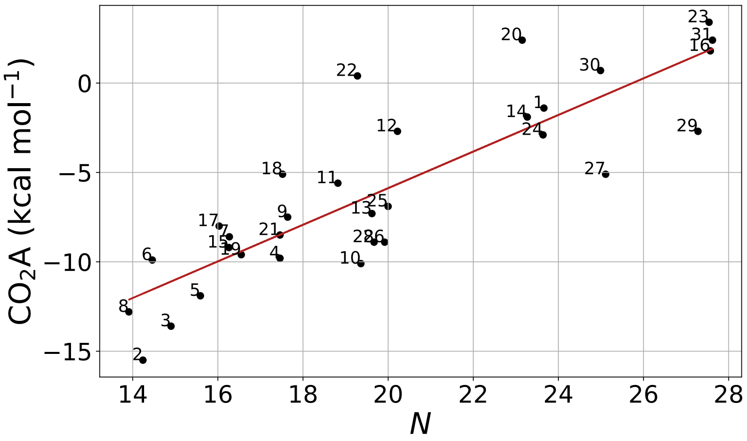

Figure S30: Correlation between Mayr's nucleophilicity parameter *N* and CO_2 affinity for 31 reactions present in the underlying dataset.

Section S9: Computational methods for target calculations (CO_2 affinity) for solvent comparison

CREST (version 3.0.2)[1,2,24] was utilized to search for the conformer ensemble for each molecule (CO_2, nucleophile, CO_2–nucleophile adduct) using the GFN2-xTB[3] method. Implicit solvation with the ALPB model[25] for DMSO and toluene was activated. To reduce the number of conformers, clustering with 50 clusters was applied. If CREST was not converged, GOAT (available in Orca 6.0.1)[26,27] was employed with the same settings. Full structure optimizations for each conformer cluster were then performed with Orca (version 6.0.1)[26,27], employing the composite method r^2SCAN-3c[28]. Implicit solvation (SMD model[13]) was employed with DMSO and toluene as the solvent. Harmonic frequency calculations at 293.15 K (for DMSO) and 353.15 K (for toluene) were conducted to confirm the convergence of the structures.

References:

[1] S. Grimme, *J. Chem. Theory Comput.* **2019**, *15*, 2847–2862.
[2] P. Pracht, F. Bohle, S. Grimme, *Phys. Chem. Chem. Phys.* **2020**, *22*, 7169–7192.
[3] C. Bannwarth, S. Ehlert, S. Grimme, *J. Chem. Theory Comput.* **2019**, *15*, 1652–1671.
[4] M. J. Frisch, G. W. Trucks, H. B. Schlegel, G. E. Scuseria, M. A. Robb, J. R. Cheeseman, G. Scalmani, V. Barone, G. A. Petersson, H. Nakatsuji, X. Li, M. Caricato, A. V. Marenich, J. Bloino, B. G. Janesko, R. Gomperts, B. Mennucci, H. P. Hratchian, J. V. Ortiz, A. F. Izmaylov, J. L. Sonnenberg, D. Williams-Young, F. Ding, F. Lipparini, F. Egidi, J. Goings, B. Peng, A. Petrone, T. Henderson, D. Ranasinghe, V. G. Zakrzewski, J. Gao, N. Rega, G. Zheng, W. Liang, M. Hada, M. Ehara, K. Toyota, R. Fukuda, J. Hasegawa, M. Ishida, T. Nakajima, Y. Honda, O. Kitao, H. Nakai, T. Vreven, K. Throssell, J. A. Montgomery Jr., J. E. Peralta, F. Ogliaro, M. J. Bearpark, J. J. Heyd, E. N. Brothers, K. N. Kudin, V. N. Staroverov, T. A. Keith, R. Kobayashi, J. Normand, K. Raghavachari, A. P. Rendell, J. C. Burant, S. S. Iyengar, J. Tomasi, M. Cossi, J. M. Millam, M. Klene, C. Adamo, R. Cammi, J. W. Ochterski, R. L. Martin, K. Morokuma, O. Farkas, J. B. Foresman, D. J. Fox, *Gaussian 16 Revision C.01*, Gaussian Inc., Wallingford (CT), United States, **2016**.
[5] A. D. Becke, *J. Chem. Phys.* **1993**, *98*, 5648–5652.
[6] P. J. Stephens, F. J. Devlin, C. F. Chabalowski, M. J. Frisch, *J. Phys. Chem.* **1994**, *98*, 11623–11627.
[7] S. Grimme, J. Antony, S. Ehrlich, H. Krieg, *J. Chem. Phys.* **2010**, *132*, 154104.
[8] S. Grimme, S. Ehrlich, L. Goerigk, *J. Comput. Chem.* **2011**, *32*, 1456–1465.
[9] F. Weigend, R. Ahlrichs, *Phys. Chem. Chem. Phys.* **2005**, *7*, 3297–3305.
[10] F. Weigend, *Phys. Chem. Chem. Phys.* **2006**, *8*, 1057–1065.
[11] B. P. Pritchard, D. Altarawy, B. Didier, T. D. Gibson, T. L. Windus, *J. Chem. Inf. Model.* **2019**, *59*, 4814–4820.
[12] M. Eckhoff, K. L. Bublitz, J. Proppe, **2024**, DOI 10.26434/chemrxiv-2024-1t4vt.
[13] J. C. Kromann, C. Steinmann, J. H. Jensen, *J. Chem. Phys.* **2018**, *149*, 104102.
[14] G. Luchini, J. V. Alegre-Requena, I. Funes-Ardoiz, R. S. Paton, *F1000Res* **2020**, *9*, 291.
[15] S. Grimme, *Chem. Eur. J.* **2012**, *18*, 9955–9964.

[16] M. Vahl, J. Proppe, *Phys. Chem. Chem. Phys.* **2023**, *25*, 2717–2728.
[17] Y. Liu, Q. Yang, J. Cheng, L. Zhang, S. Luo, J.-P. Cheng, *ChemPhysChem* **2023**, e202300162.
[18] J. P. Perdew, K. Burke, M. Ernzerhof, *Phys. Rev. Lett.* **1996**, *77*, 3865–3868.
[19] E. D. Glendening, C. R. Landis, F. Weinhold, *J. Comput. Chem. Chem.* **2019**, *40*, 2234–2241.
[20] R. Ditchfield, *Molecular Physics* **1974**, *27*, 789–807.
[21] Jacot-Descombes, Lauriane, Turcani, Lucas, Jorner, Kjell, **n.d.**
[22] F. Pedregosa, G. Varoquaux, A. Gramfort, V. Michel, B. Thirion, O. Grisel, M. Blondel, P. Prettenhofer, R. Weiss, V. Dubourg, J. Vanderplas, A. Passos, D. Cournapeau, M. Brucher, M. Perrot, É. Duchesnay, *Journal of Machine Learning Research* **2011**, *12*, 2825–2830.
[23] H. Mayr, M. Patz, *Angew. Chem. Int. Ed.* **1994**, *33*, 938–957.
[24] P. Pracht, S. Grimme, C. Bannwarth, F. Bohle, S. Ehlert, G. Feldmann, J. Gorges, M. Müller, T. Neudecker, C. Plett, S. Spicher, P. Steinbach, P. A. Wesołowski, F. Zeller, *J. Chem. Phys.* **2024**, *160*, 114110.
[25] S. Ehlert, M. Stahn, S. Spicher, S. Grimme, *J. Chem. Theory Comput.* **2021**, *17*, 4250–4261.
[26] F. Neese, F. Wennmohs, U. Becker, C. Riplinger, *J. Chem. Phys.* **2020**, *152*, 224108.
[27] F. Neese, *WIREs Comput Mol Sci* **2022**, *12*, e1606.
[28] S. Grimme, A. Hansen, S. Ehlert, J.-M. Mewes, *J. Chem. Phys.* **2021**, *154*, 064103.
